# Stress-resistant but phage-sensitive host mutants induced by phage T4 ghost adsorption

**DOI:** 10.3389/fmicb.2025.1683709

**Published:** 2025-10-10

**Authors:** Takehiko Kenzaka, Katsuji Tani

**Affiliations:** ^1^Department of Life Science, Faculty of Science and Engineering, Setsunan University, Neyagawa, Japan; ^2^Environmental Science and Microbiology, Faculty of Pharmacy, Osaka Ohtani University, Tondabayashi, Japan

**Keywords:** phage ghost, mutant, phage, *Escherichia coli*, diversity

## Abstract

The main effect of lytic phages on the host is cell lysis, and genetic impact of short-term contact between the host and the phage remains unknown unless genetic exchange occurs. In this study, we found that the adsorption of a lytic phage to the host cell can rapidly alter the genetic and physiological properties of hosts that have escaped lysis without relying on gene transfer. After adsorption of the lytic phage T4 to *Escherichia coli* (host) cells, 1% of *E. coli* cells exhibited an approximately 85-fold increase in spontaneous mutant frequency, which was measured based on antibiotic resistance. Phage ghosts increased the incidence of mutator strains exhibiting elevated expression of the error-prone DNA polymerase IV gene (*dinB*), while the emergence of mutators was suppressed in the *dinB*-deficient strain. Adsorption of ghosts onto the cell surface triggered global changes in gene expression in surviving cells, including upregulation of DNA polymerase IV. This upregulation led to mutations in host genes such as *tfaR* and *marR*, which were associated with high mutant frequency. Phage- or ghost-derived mutator strains showed a higher frequency of resistance to reactive oxygen species and acid stresses than the parental strain but a lower frequency of resistance to phage T4. These findings suggested that phage ghosts may promote host cell survival and alter their physiological characteristics, thus contributing to the production of progeny virions in future phage attacks.

## 1 Introduction

Phages are ubiquitous and are the most abundant biological entities on Earth (Bergh et al., 1989; Fuhrman, 1999). Lytic phages replicate within their host cells and must burst them open to enable transmission to the next generation. Phage-induced lysis influences bacterial species distribution, biodiversity, and particle size distribution (Proctor and Fuhrman, 1990; Paul, 2008). Bacteria have evolved resistance to phage attack via several mechanisms (Koskella and Brockhurst, 2014). Bacterial resistance to phages is frequently derived from *de novo* mutations that modify the structure of surface molecules targeted by phages, thereby preventing phage attachment. In a study that examined the long-term coexistence of phages with hosts for 200 generations, the hosts generated a significant proportion of mutator strains with mutant frequencies higher than their ancestral strains (Pal et al., 2007). Phages are thus thought to promote mutations in the host, but the molecular mechanisms of the processes within host cells during and after phage attack remain unknown. Phage infectivity requires the evolution of the tail fiber on the phage. Because phages cannot change their nucleotide sequences by themselves, the evolution of the tail fiber occurs during their propagation within host cells.

This study hypothesized that phages can proactively alter host cell characteristics to enhance the probability of producing progeny virions during future attacks. The potential short-term mechanisms include: (i) altering the mutation frequency of host cells, (ii) increasing phage sensitivity in surviving host cells to facilitate lysis in subsequent attacks, and (iii) ensuring that not all host cells are killed, allowing some to survive and propagate for future infections.

To test these hypotheses, we investigated the fate of the host after an attack by a lytic phage for a short period (10 min) using laboratory populations of *Escherichia coli* and the phage T4 system. We examined the mutant frequency in host cells that escaped lysis by screening mutator strains that emerged after the attack, as well as their genetic, transcriptional, and physiological properties, such as resistance to phage, reactive oxygen species (ROS), and acidity. We found that the adsorption of a phage ghost to the host cell surface increased the mutant frequency of the bacterium without engaging previously known mechanisms of mutagenesis, such as the transfer of genetic material. Phage ghosts, which possess only the phage protein coat, are naturally produced during phage propagation. We provided the possible mechanisms by which phage ghosts promote host cell survival and alter their physiological characteristics, which may contribute to the production of progeny virions in future phage attacks.

## 2 Materials and methods

### 2.1 Bacterial strains, phage strains, and culture conditions

*E. coli* K-12 wild-type strain W3110 (Bachmann, 1972) and the mutator strains, and NBRC 13168 (Hill and Simson, 1961) were grown until mid-log phase (4 h) or stationary phase (overnight) in Luria–Bertani broth (1% tryptone, 0.5% yeast extract, 0.5% NaCl) at 37 °C. *E. coli* phages T4 NBRC 20004 was propagated overnight with *E. coli* strains W3110 or 13168 in 1 L of LB broth, containing 0.2% MgSO_4_ and 10 mmol/L CaCl_2_, at 37 °C. The purification of dsDNA phages by ultracentrifugation was done using the previously described procedure (Kenzaka et al., 2007) to exclude the possibility of host cell component effect. After purification by ultracentrifugation, the plaque forming units (PFU) of phage were measured with *E. coli* W3110 using the soft-agar overlay method (Kenzaka et al., 2007).

### 2.2 Preparation of phage ghosts and UV-irradiated phages

The ghosts of phage T4 were prepared by the osmotic shock method with sodium acetate (Duckworth, 1971). Briefly, ghosts were prepared by the addition of 2 volumes of 3 M sodium acetate to 1 volume of phage T4 which were about 10^11^ phage/mL. Equilibration of the phage in the salt solution at 0 °C for 15 min was followed by rapid dilution into 100 volumes of cold distilled water. DNase treatment with 3 U RNase-Free DNase (Promega Japan, Tokyo, Japan) was performed at 37 °C for 30 min. The ghosts of phage T4 were also recovered from supernatant fraction after ultracentrifugation for the purification of dsDNA phages. The PFU of phage ghosts decreased to 0.1% by the preparation.

UV irradiation with a peak wavelength of 254 nm was performed in phage suspension (about 10^11^ phage/mL) using 15-W germicidal lamps National GL-15 (Panasonic Co., Kadoma, Japan) at a distance of 30 cm from the light source to sample for 15 min. The PFU of phage ghosts and UV-irradiated phages were measured with *E. coli* W3110 using the soft-agar overlay method (Kenzaka et al., 2007) instead of using an intact phage. The particle numbers of intact phage, phage ghosts and UV-irradiated phages were also measured by staining with SYBR gold and Qubit Protein Reagent (ThermoFisher Scientific, Paisley, UK).

### 2.3 Phage or ghost attack experiments

A part of the frozen cultures from five independent single colonies of *E. coli* W3110 was inoculated into 10 mL of aerobic LB broth and incubated at 37 °C overnight. The diluted culture (about 10^3^ cells; cell number of nalidixic acid resistant mutant was negligible) was transferred to fresh 10 mL of LB broth and incubated at 37 °C overnight. The stationary phase *E. coli* culture was washed and resuspended in fresh LB broth. One milliliter of diluted *E. coli* culture (1 × 10^9^ CFU/mL) was incubated with phage T4 at 37 °C for 10 min at a multiplicity of infection (MOI) of 1 or 5. In order to minimize the impact of unadsorbed phages and daughter phages generated during cultivation, the mixture was immediately diluted 1:1,000,000 and plated on LB agar plates. Approximately 100 cells and phages were present on each agar plate. The plates were incubated at 37 °C for 2 days.

The number of initial hosts was determined as CFU before phage attack experiments. The lysis rate after the attack by phage T4 was determined by comparing the number of initial host colonies and colonies that escaped lysis. In our experiments, 30–120 colonies were formed per LB agar plate. One hundred sixty colonies that escaped lysis were randomly selected in six independent experiments and subjected to further treatment. To examine the effect of the phage ghost, it was incubated with the host as described above instead of using an intact phage. Since ghost preparation by the osmotic shock method had diluted the phage suspension and reduced PFU of phage, the ratio of phage ghosts to bacteria was not calculated with actual PFU but adjusted to the theoretical ratio same as that before ghost preparation to examine the effect of same number of phage particles on bacteria. The particle numbers of intact phage, phage ghosts and UV-irradiated phages were also confirmed using SYBR gold and Qubit Protein Reagent staining. Initial *E. coli* number was normalized to 1 × 10^9^ CFU/mL.

### 2.4 Presence of viable phages in a colony

The randomly selected colonies that escaped lysis after the addition of phage T4 or the ghost were inoculated in LB broth and incubated at 37 °C overnight. Mitomycin C (1 μg/mL) was added to promote host lysis, and the cultures were further incubated at 37 °C overnight. Five microliters of the culture supernatant were spotted on LB agar after 100 μL of *E. coli* W3110 culture was plated on LB agar. After incubation at 37 °C overnight, plaque formation was observed.

### 2.5 Mutant frequency

The randomly selected colonies that escaped lysis after the addition of phage T4 or the ghost were inoculated in LB broth and incubated at 37 °C overnight. Mutant frequencies (frequency of antibiotic resistant mutants) were determined in triplicate at a minimum in LB agar medium with and without 10 μg/mL of nalidixic acid or 10 μg/mL of streptomycin. The colonies that showed mutant frequencies above 10^−7^ after the phage attack experiments were designated as mutator strains 1–13, 1–14, 2–32, 4–9, and 4–29. The five phage-derived mutator strains were obtained after three independent phage attack experiments. The colonies that showed mutant frequencies above 10^−7^ after the ghost attack experiments were designated as mutator strain g62, g74, g94, gw25, and gb37. The five ghost-derived mutator strains were obtained after three independent ghost attack experiments. The overnight cultures in LB broth were frozen at −80 °C and were set at zero generation. Sixteen populations of each mutator mutant were inoculated into 16 tubes with fresh LB broth and incubated at 37 °C overnight. The populations were used to examine the fluctuation of elevated mutant frequencies of nalidixic acid resistance.

In order to confirm the maintenance of elevated mutant frequencies over bacterial generations, 10 μL of each cultured mutator was transferred to 10 mL of fresh LB broth every 24 h for 10 transfers (approximately 100 generations). Cultures were frozen at −80 °C after every second transfer.

In order to exclude the possibility of a preexisting mutator, which might be present prior to exposure to the phage or ghost, the following experiment was performed. A part of the frozen culture from a single colony of *E. coli* W3110 (prior to phage exposure) was inoculated into LB broth and incubated at 37 °C overnight. Twenty populations of the diluted culture were transferred to fresh LB broth and incubated at 37 °C overnight. The culture was plated on LB agar plates and incubated at 37 °C for 2 days. From each plate, 20–40 colonies were randomly chosen and used to determine the mutant frequency. A total of 893 colonies were used to examine the presence of a preexisting mutator.

### 2.6 Preparation of low molecular weight fraction from damaged cells

Effect of low molecular weight fraction of the particle-free supernatant made from damaged cells after phage or ghost attack experiments on emergence of mutator strain was examined. After one milliliter of diluted *E. coli* culture (approximately 1 × 10^9^ CFU/mL) was incubated with phage T4 or the ghost at 37 °C for 10 min in phage or ghost attack experiments, the mixture of cell and phages or ghosts was centrifuged and the supernatant was filtrated with Whatman Anotop 25 mm Syringe Filter (0.02 μm pore size; GE Healthcare Life Sciences, Buckinghamshire, England) and Amicon Ultra-4100 K devices (Merck KGaA, Darmstadt, Germany) to recover 100 kDa NMWL substances released from the damaged cells. The particle-free supernatant was immediately subjected to further experiments.

The stationary phase *E. coli* culture was washed and resuspended in fresh LB broth. The *E. coli* culture (approximately 1 × 10^9^ CFU/mL) was mixed in the particle-free supernatant containing the low molecular weight fraction and incubated at 37 °C for 10 min. The mixture was diluted 1:1,000,000 and plated on LB agar plates. The plates were incubated at 37 °C for 2 days. Mutant frequency of the randomly selected 300 colonies was examined as described above.

### 2.7 Phage resistance spot test

The mutator strains were inoculated in LB broth and incubated at 37 °C overnight. One hundred μL of the culture was plated on LB agar, and then five microliters of the Phage T4 suspension was spotted on the LB agar. After incubation at 37 °C overnight, plaque formation was observed.

### 2.8 Scanning electron microscopy (SEM)

One milliliter of diluted *E. coli* culture was incubated with either phage T4 or phage ghosts at 37 °C for 5 min. The mixtures were then fixed in 4% paraformaldehyde and 1% glutaraldehyde in 0.1 M sodium cacodylate buffer for 2 h. After fixation, portions of each sample were filtered through 25-mm-diameter Whatman ANODISC membrane filters with a pore size of 0.02 μm (Global Life Sciences Technologies Japan K. K., Tokyo, Japan), followed by washing with 0.1 M sodium cacodylate buffer. The filters were placed on filter paper soaked in 0.1 M sodium cacodylate buffer, post-fixed for 1 h with 1% osmium tetroxide buffer, and washed again with 0.1 M sodium cacodylate buffer. For dehydration, the filters were sequentially placed on filter paper soaked in 50, 80, and 100% ethanol, and then in 50, 80, and 100% *tert*-butyl alcohol. The samples were subsequently freeze dried and coated with gold. Finally, the gold-coated samples were imaged using a JSM 6510LA scanning electron microscope (JEOL Ltd., Tokyo, Japan) at 15 kV.

### 2.9 DNA extraction and purification

*Escherichia coli* DNA was extracted using the QIAamp DNA Isolation Kit (Qiagen KK, Tokyo, Japan), according to the modified procedure described in the instruction manual. The cell suspension was mixed with 15 mg/mL of lysozyme solution and was incubated at 37 °C for 1 h. DNA extraction was then performed. Phage DNA was extracted using the lambda phage DNA Isolation kit (Qiagen).

### 2.10 Gene deletion strain

For the construction of the gene (*dinB* [DNA polymerase IV], *marR* [transcriptional repressor MaR], and *tfaR* [tail fiber assembly protein])- deficient mutant strain, the Quick & Easy *E. coli* Gene Deletion Kit (Gene Bridges GmbH, Heidelberg, Germany) was employed according to the protocol recommended by the technical manual. Briefly, *E. coli* W3110 was transformed with plasmid pRedET (Gene Bridges GmbH), and a linear DNA fragment FRT-flanked kanamycin resistant cassette with 50-mer homologous sequences for target gene was generated by PCR with the target primer set. Red/ET recombination protein was induced, was transformed with the above PCR fragment. The FRT-flanked kanamycin resistant cassette was recombined on the target gene. The kanamycin selection marker was removed using the plasmid 707-FLPe (Gene Bridges GmbH). The gene disruption was verified by colony PCR followed by DNA sequencing. The detailed protocol was described in a Supplementary file.

### 2.11 *dinB*–expression plasmid construction

A 1,565 bp fragment of *E. coli* W3110 chromosomal DNA, including *dinB* and its promoter and operator region (8094–9,653 region in GenBank entry D38582), was amplified by PCR with *dinB* region primers (dinB8094f, 5’-ggagctcgcgccagtg-3’ and dinB9653r, 5’-gatgcatacagtgataccctcata-3’). The PCR product was inserted into the pGEM-T-Easy Vector System (Promega), according to the manufacturer’s instructions. pGEM-T:*dinB* was introduced into *E. coli* W3110 by electroporation at 3 kV.

### 2.12 Genome sequencing of mutator strains

The genomes of *E. coli* in our laboratory were sequenced using GridION (Oxford Nanopore Technologies) sequencing technique provided by the Bioengineering Lab. Co., Ltd. (Sagamihara, Kanagawa, Japan). The assembly of the sequencing reads yielded one contig. In addition, the whole genomes of *E. coli* strains were sequenced by the 100 bp sequencing on the Illumina Hiseq 2000 sequencing system provided by the Hokkaido System Science Co., Ltd. (Sapporo, Hokkaido, Japan). The Illumina Hiseq 2000 sequencing run yielded from 11 to 17 million high-quality filtered reads with 101 bp reads sequencing. High quality reads were mapped to the one chromosome constructed by the GridION system as reference using CLC Genomics Workbench v6.5 (CLCbio, Cambridge, MA, USA). The unmapped sequences were assembled into contigs using CLC Genomics Workbench v6.5, and subjected to Nucleotide BLAST for Enterobacteria phage T4 (GenBank accession number AF158101). Single nucleotide polymorphisms (SNPs) and small insertions/deletions among mutator strains were examined using CLC Genomics Workbench v6.5. The annotation for the location of SNPs was manually performed by the database Profiling of *E. coli* chromosome^1^ and Protein Details for strain W3110 on NCBI^2^.

### 2.13 Mutant gene–expression plasmid construction

Fragments of *E. coli* W3110 and ghost-derived mutator strains chromosomal DNA, including mutant genes on the mutator strains and their promoter and operator regions (4383568–4,387,273 region in GenBank entry AP009048 for *frdD*, 4,459,126–4,460,613 region for *fbp*, 1,034,432–1,042,007 region for *flgG*), were amplified by PCR with the region primers (frdD-region-f [5’-agcaaatgtggagcaagagg-3’] and frdD-region-r [5’-acggcgagacaaattttacg-3’] for *frdD*, fbp-region-f [5’-gagttcctggtggacgaaaa-3’] and fbp-region-r [5’-atcagctcaatgccttgctt-3’] for *fbp*, flgG-region-f [5’-cgacccattttgcgtttatt-3’] and flgG-region-r [5’-cctgcgccataatagtggtt-3’] for *flgG*). The PCR products were inserted into the pGEM-T-Easy Vector System (Promega), according to the manufacturer’s instructions. The constructed plasmids were introduced into *E. coli* W3110 by electroporation at 3 kV. The same direction of inserted fragments on the plasmid was confirmed by sequencing.

### 2.14 Frequencies of phage-resistant mutants

To determine the mutant frequencies of phage resistance, 10 isolated colonies of W3110, W3110 *dinB*-deficient mutant, W3110 carrying the plasmid described below, and phage- or ghost- derived mutator strains were inoculated into 10 tubes with LB broth, grown with agitation until approximate concentration of 10^9^ CFU/mL and diluted in 10-fold increments. One hundred μL aliquots from the 10^−2^–10^−3^ dilutions were mixed with 10 mL soft agar (0.7%) and 100 μL phage suspension (10^8^ particles per plate for phage T4, MOI of 100–1,000) and poured on LB agar plates. The plates were incubated at 37 °C for 5 days because some of the phage-resistant mutants grow very slowly. Simultaneously, one hundred microliters aliquots of 10^−6^ dilutions were plated on LB agar without phage for determination of CFU numbers. To calculate mutation frequencies of phage resistance, numbers of mutants in one mL of culture were divided by total numbers of live bacterial cells and shown as the median of mutant frequencies.

### 2.15 Phage adsorption assay

Phage adsorption assay was done using the previously described procedure (Kropinski, 2009). *E. coli* W3110 and ghost-derived mutator strains were grown in LB broth to exponential phase, and then infected with the phage T4 at MOI 0.001, and incubated at 37 °C. The 0.05 mL of supernatants were taken at different time intervals such as 1, 5, and 10 min, and diluted in chilled LB broth with drops of chloroform. The non-adsorbed phages were titered on *E. coli* NBRC13168 using the soft-agar overlay method. The experiment was repeated three times independently.

### 2.16 Oxidative stress resistance assay

Four to five colonies for each mutator strain were pooled in LB agar plate after they were resuscitated from glycerol stock. The overnight culture was diluted 1:100 in fresh LB broth and grown at 37 °C. Exponentially growing cells (OD_600_ = 0.4) were obtained from cultures grown for 4 h. To assay oxidative stress resistance, H_2_O_2_ was added to the cultured at final concentration of 50 mmol/L and incubated for 15 min at 37 °C. After H_2_O_2_ exposure, cells were serially diluted in phosphate buffered saline (PBS) and plated on LB agar plates. The number of viable colonies was determined to calculate the CFU/mL. The survival rate was calculated based on the ratio of CFU of cells after H_2_O_2_ exposure to CFU before the H_2_O_2_ exposure. The experiment was repeated five times independently.

### 2.17 Acid stress resistance assay

Acid resistance assay was performed referring the previously described procedure (Richard and Foster, 2004). Four to five colonies for each mutator strain were pooled in LB agar plate after they were resuscitated from glycerol stock. The overnight culture was diluted 1:100 in fresh LB broth and grown at 37 °C. Exponentially growing cells were obtained from cultures grown for 4 h. To assay acid resistance, sample cells were diluted 1:100 with M9 minimal media supplemented with 0.4% glucose and 1.5 mmol/L glutamate, which was previously adjusted to pH 2.5 with concentrated HCl and incubated for 15 min at 37 °C. After acid exposure, cells were serially diluted in PBS and plated on LB agar plates. The number of viable colonies was determined to calculate the CFU/mL. The survival rate was calculated based on the ratio of CFU of cells after acid exposure to CFU before the acid exposure. The experiment was repeated five times independently.

### 2.18 Competition experiments

The pairs of W3110 and W3110 strain, W3110 and g62 strain, W3110 and gw25, W3110 and gb37 that carried identical *gyrA* or *rpoB* mutation, were subjected to competition experiments because different nalidixic acid/rifampicin resistance mutations have specific fitness benefits (Katz and Hershberg, 2013).

About 30,000–60,000 unmarked W3110 cells were spotted on a sterile polycarbonate filter membrane. Since mutant frequencies of nalidixic acid and rifampicin resistance in W3110 were <10^−8^, no Rif^R^ or Nal^R^ mutants were present in the initial inoculum. Then a mixture of 100–200 marked W3110 cells (marked with either Nal^R^ or Rif^R^) and 100–200 marked ghost-derived mutator cells (marked with either Rif^R^ or Nal^R^) were spotted on the same filter.

The exact ratio of unmarked, nalidixic acid- or rifampicin- resistant cells inoculated onto the filter was estimated through live counts (day 0). Then the ratio of marked ghost-derived mutator to marked W3110 was measured after 2 days. The measurement of these ratios was done by scraping the mixture spots, placing them in PBS. Then 50 μL from the suspension were plated on LB agar containing 100 μg/mL rifampicin or 10 μg/mL nalidixic acid, and viable cells were counted the following day.

The results with the marked W3110 cells were used to normalize the measured competitive index (CI) for Rif^R^-marked mutator versus Nal^R^-marked W3110 strains. Three replicates of each competition experiment were performed. The CI was calculated for each Rif^R^ or Nal^R^ -marked ghost-derived mutator from the change in the Nal^R^ or Rif^R^ /W3110 ratio from Day 0 to Day 2. The more detailed protocol was described in a Supplementary file.

### 2.19 *GyrA* sequencing

The main target gene for resistance to nalidixic acid is *gyrA*. To know the mutations that confer the obtained resistances, *gyrA* was amplified and then sequenced. The primers used to amplify the portion of the *gyrA* encoding the main set of mutations conferring resistance to nalidixic acid were: 5’-TACACCGGTCCACATTGAGG-3’ and 5’-TTAATGATTGCCGCCGTCGG-3’ (Trindade et al., 2009). The same primers were used for sequencing straight from the PCR product.

### 2.20 Quantitative reverse transcription PCR

To examine the mRNA expression of *dinB* in *E. coli*, the stationary phase *E. coli* culture grown in LB broth was washed and resuspended in fresh LB broth. A quantity of 1 mL of diluted *E. coli* culture (1 × 10^9^ CFU/mL) was incubated with phage T4 or the ghosts at 37 °C for 5 min at an MOI of 1 (ratios of phage ghosts or UV-irradiated phages to bacteria were same as those before ghost and UV irradiation preparation). In order to exclude the possibility that the buffer for phage ghost treatment induced the mRNA expression of *dinB* in *E. coli*, the *E. coli* culture was incubated with the same buffer for ghost treatment without active phage or ghosts at 37 °C for 5 min.

To examined the effect of particle-free supernatant derived from phage or ghost attack experiments on the *dinB* mRNA expression, the host was suspended in the particle-free supernatants and incubated at 37 °C for 5 min. The particle-free LB medium was used as a negative control.

To examine the mRNA expression of *dinB* in mutator and parent strains under normal condition, the mid log phase *E. coli* culture grown in LB broth was washed and resuspended in fresh LB broth.

To validate the results of microarray assay of mutator strains described above, exponentially growing cells of *E. coli* W3110 and the three ghost-derived mutator strains (g62, gw25, gb37) were obtained from cultures grown in LB broth at 37 °C for 4 h.

Total RNA was extracted using the RNeasy Mini Kit (Qiagen). First-strand cDNA was prepared with the PrimeScript RT reagent Kit (Perfect Real Time) (Takara Bio Inc.). The reverse transcription reactions were performed with 0.1 μmol/L gene specific reverse primer mix (Supplementary Table 1) at 42 °C for 15 min followed by incubation at 80 °C for 5 s for inactivation of enzyme. Primer sets were designed in this study except for primer set for 16S rRNA gene (Arunasri et al., 2013).

Duplicate 20 μL PCR reactions were performed for each cDNA, and comprised 0.5 μL of cDNA, 1 x PowerUp™ SYBR® Green Master Mix (ThermoFisher Scientific, Paisley, UK), and 0.5 μmol/L of forward and reverse primers (Supplementary Table 1). The PCR reaction mixture was pre-incubated at 50 °C for 2 min for UDG activation and at 95 °C for 2 min for Dual-Lock™ Taq DNA polymerase activation, and subjected to 40 cycles under the following thermal conditions: 95 °C (15 s) and 60 °C (60 s). Relative expression of genes was calculated by ΔΔCT method which is based on product cycle threshold (CT). Expression of 16S rRNA gene was used as an internal standard for RT-PCR. All values reported represent the mean of at least four independent experiments.

### 2.21 Microarray analysis

For a global transcriptome analysis of mutator strains under normal condition without phage or ghost, exponentially growing cells of *E. coli* W3110 and the three ghost-derived mutator strains (g62, gw25, gb37) were obtained from cultures grown in LB broth at 37 °C for 4 h. For a global transcriptome analysis of host cells following an attack by phage T4 or phage ghost, *E. coli* W3110 cells after incubated with phage T4 or the phage ghost at 37 °C for 10 min were obtained in phage or ghost attack experiments. To mask the effect of the SM buffer, the host was incubated with the SM buffer as described above instead of using the phage T4 or the ghost. The experiments on the *E. coli* W3110 cells incubated with the SM buffer was used as control.

Total RNA was extracted using the RNeasy Mini Kit (Qiagen). Extracted RNAs from three independent cultures were mixed. Microarray hybridizations were performed at Hokkaido System Science Co., Ltd. according to the manufacturer’s protocol, using the workflow for one-color *E. coli* Gene Expression Microarray, 8x15K (Agilent Technologies, Santa Clara, CA, USA). Each microarray was composed of 15,208 probes representing the complete genome of four *E. coli* strains: K-12-MG1655, O157: H7 VT2-Sakai, CFT073, and EDL933. The microarray slides were scanned and the gene expression profiles analyzed at Hokkaido System Science Co., Ltd., according to the manufacturer’s protocol. The following filters were applied to improve the quality of the data: eliminate saturated signal, eliminate the nonuniformity of the background, eliminate the nonuniformity of features, Feature Population Outlier, and eliminate the low signal features of the background signal. The data were normalized using 75 percentile shift normalization using Genespring (Agilent Technologies). The transcription data for all genes was expressed as the ratio of the mutator signal to the W3110 signal (ArrayExpress accession E-MTAB-5636). Z score for each gene set was calculated as follows (Kim and Volsky, 2005). First, from input data containing fold change values for each gene between two experimental groups, mean of total fold change values (*μ*) and standard deviation of total fold change values (*δ*) of a given microarray data set were calculated. Then, when the mean of fold change values of genes for a given gene set was Sm and the size of a given gene set was m, the Z score was calculated as Z = (Sm –μ)*m^1/2^ / δ.

### 2.22 Statistics

The Mann–Whitney U test was performed to compare phenotypic and transcriptional characterization of mutator strains using the Bell Curve Excel Statistics software v3.2 (SSRI Co., Ltd., Tokyo, Japan). Rate of emergence of mutator were compared using relative risk and the 95% confidence interval, Fisher’s exact test with Bell Curve Excel Statistics software v3.2.

## 3 Results

### 3.1 Mutant frequency of *Escherichia coli* after attack by phage T4

**Figure 1:**
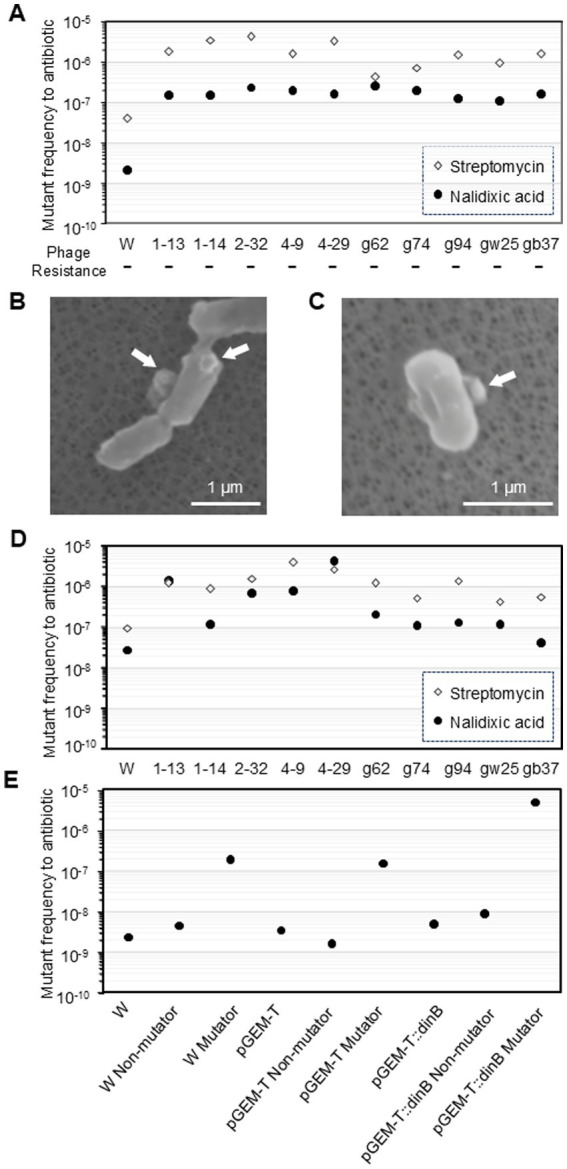
Characteristics of phage T4 or the ghost-derived mutator strains. Mutant frequency to nalidixic acid and streptomycin at 0 generation was shown as median of 16 independent cultures. −, negative in phage resistance spot test **(A)**. After *E. coli* cells were incubated with the phage or the ghost for 5 min, samples were fixed and the adsorption of the phage **(B)** and the ghost **(C)** to the cells was observed by scanning electron microscopy. Arrows indicate phage or ghost. Mutant frequency to nalidixic acid and streptomycin at 100 generation **(D)** and mutant frequency to nalidixic acid in *dinB*-overexpression strain were shown as median of 16 independent cultures **(E)**.

To test whether a short-term attack by lytic phages affects the physiological properties of host cells, we examined the fate of *E. coli* after the phage T4 attack (Table 1). Approximately 62 and 67% of the initial hosts were lysed at MOI of 1 and 5, respectively. Additionally, 8.4 and 3.4% of the colonies contained viable phages, indicating that both intact and phage-infected cells coexisted in a single colony. Phages were maintained in a colony of the bacterial strain by infecting susceptible variants of the strain. Approximately 29 and 28% of the colonies contained no viable phages and thus completely escaped phage-induced lysis. The mutant frequency of nalidixic acid and streptomycin resistance in the colonies that escaped lysis was then examined. In the strain W3110, the median mutant frequencies of nalidixic acid resistance and streptomycin resistance were 2.1 × 10^*−*9^ and 4.0 × 10^*−*8^, respectively (Figure 1A).

**Table 1:** Fate of *E. coli* after attack by phage T4 or ghost.

Addition of phage or ghost ^a^	MOI	Lysis ^c^ CFU/mL	Phage (+) colony CFU/mL	Non-mutator CFU/mL	Mutator ^d^ CFU/mL	Relative risk [95% CI] *p*-value
None	-	-	-	1 × 10^9^	<1.1 × 10^6^ (<0.11%)	-
T4 phage	1	6.2 × 10^8^ (62%)	8.4 × 10^7^ (8.4%)	2.9 × 10^8^ (29%)	1.2 × 10^7^ (1.2%)	>11 [1.4–82.2<] 0.004
T4 phage	5	6.7 × 10^8^ (67%)	3.4 × 10^7^ (3.4%)	2.8 × 10^8^ (28%)	1.2 × 10^7^ (1.2%)	>11 [1.4–82.2<] 0.004
T4 ghost [osmotic]	1	2.0 × 10^8^ (20%)	<6.3 × 10^6^ (<0.06%)	7.2 × 10^8^ (72%)	8.0 × 10^7^ (8.0%)	>71 [9.0–565<] < 0.001
T4 ghost [osmotic]	5	6.7 × 10^8^ (67%)	<6.3 × 10^6^ (<0.06%)	3.1 × 10^8^ (31%)	3.4 × 10^7^ (3.4%)	>29 [3.9–208<] < 0.001
T4 ghost [supernatant]	1	1.4 × 10^8^ (14%)	<6.3 × 10^6^ (0.06%)	8.1 × 10^8^ (81%)	5.4 × 10^7^ (5.4%)	>45 [5.3–378<] < 0.001
▵*dinB* ^b^ + T4 phage	1	7.4 × 10^8^ (74%)	1.1 × 10^8^ (11%)	1.5 × 10^8^ (15%)	<4.5 × 10^5^ (<0.045%)	-
▵*dinB* ^b^ + T4 ghost [osmotic]	1	3.1 × 10^8^ (31%)	<6.3 × 10^6^ (<0.06%)	6.9 × 10^8^ (69%)	1.8 × 10^6^ (0.18%)	2.3 [0.14–37] 0.51
pGEM-T:*dinB* + T4 ghost [osmotic]	1	1.9 × 10^8^ (19%)	<6.3 × 10^6^ (<0.06%)	8.1 × 10^8^ (81%)	7.0 × 10^7^ (7.0%)	>62 [7.8–503<] < 0.001

a T4 phage or the ghost were mixed with *E. coli* W3110 culture (1 × 10^9^ CFU/mL) for 10 min, and plated on LB agar. The ghost was prepared by osmotic method [osmotic] or ultracentrifugation [supernatant].

b T4 phage or the ghost were mixed with *E. coli* ▵*dinB* culture.

c Number of lysis colony were calculated by subtracting number of survived colony from number of initial colony.

d Mutator showed mutant frequency to nalidixic was over 10^−7^.

In contrast, 1.2% of the initial hosts (1.2 × 10^7^ CFU/mL) had mutant frequencies above 10^−7^ after phage attack at MOI of both 1 and 5, which is a 10 to 100-fold increase. None of the colonies had median mutant frequencies of nalidixic acid resistance above 10^−7^ (<1.1 × 10^6^ CFU/mL, <0.11% of the total colonies) in population without phage exposure using a total of 893 colonies derived from 20 liquid cultures (Table 1). Consequently, the relative risk in phage exposure was >11, and 95% confidence interval was 1.40 to 82.2 < (*p* < 0.004). Since the lower limit of the 95% confidence interval is greater than one, phage exposure was thought to be associated with emergence of mutator.

Since the mutator strains were able to escape lysis following phage T4 infection, it might be possible that they possessed fewer phage receptors. However, a phage resistance spot test with fresh phage T4 showed that all mutator strains (1–13, 1–14, 2–32, 4–9, and 4–29) were not resistant to phage T4 (Figure 1A). These results indicated that attack by lytic phages, even with short-term exposure, affected the mutant frequency of host cells that escaped phage-induced lysis.

### 3.2 Effect of phage ghosts on the emergence of mutator strains

The mechanism responsible for the emergence of mutator strains after attack by lytic phage T4 for a short period was investigated. Phage T4 ghosts were prepared by the osmotic shock method, and ghost attack experiments were performed. Phage ghosts, composed exclusively of the phage protein coat without any genetic material, have various biological effects on cells (Duckworth, 1970). The phage ghost, like the phage, was confirmed to adsorb to the host cell using SEM (Figures 1B,C). After the attack by phage T4 ghosts at MOI of 1 and 5, approximately 20 and 67% of the initial host colonies were lysed (Table 1), and 80 and 34% escaped lysis, respectively. The percentage of mutator strains significantly increased to 8 and 3.4% at MOI of 1 (relative risk, >71; 95% confidence interval, 9.0 to 565<; *p* < 0.001) and MOI of 5 (relative risk, >29; 95% confidence interval, 4 to 208<; *p* < 0.001), respectively. T4 phage ghosts which were recovered from the ultracentrifugation supernatant fraction, were also used to perform ghost attack experiments. The percentage of mutator strains increased to 5.4% (relative risk, >45; 95% confidence interval, 5.3 to 378<; *p* < 0.001) (Table 1).

To examine whether extracellular substances derived from cells damaged by phage T4 or ghost attack affect the emergence of mutator strains, the low molecular weight fraction of the particle-free supernatant made from damaged cells was exposed to intact cells, and their response was examined. No mutator strain was detected at least at a rate of 0.3% that of the initial hosts (<3.0 × 10^6^ CFU/mL).

The median mutant frequencies in ghost-derived mutator strains (g62, g74, g94, gw25, and gb37) were also significantly higher than the W3110 parental strain (Figure 1A). Therefore, the emergence of mutator strains might have been due to the presence of ghosts in the phage population; thus, it was independent of phage or bacterial DNA entry and extracellular substance derived from damaged cell. The high mutant frequency in the mutators was maintained even after 100 generations (Figure 1D).

### 3.3 Role of DNA polymerase IV in the emergence of mutator strains and phage resistance

To explore the molecular mechanism by which ghosts increase mutant frequency after adsorption, the effects of DNA polymerase IV on mutant frequency were examined. Error-prone DNA polymerase IV is induced by the SOS response in bacteria and is responsible for stress-induced mutagenesis (Mckenzie et al., 2001; Ponder et al., 2005). The degradation of phage DNA is known to induce the SOS response in *Salmonella typhimurium* (Barbe et al., 1983), but the relationship between ghost adsorption and DNA polymerase IV induction remains unknown.

The contribution of DNA polymerase IV to the frequency of emergence of mutator was examined (Table 1). When the *dinB*-deficient strain was used, no mutator strain was detected among the 576 isolates that escaped lysis after the addition of phage T4 (<4.5 × 10^5^ CFU/mL). One mutator strain was detected among the 384 isolates that escaped lysis after the addition of T4 ghosts (0.18% of the initial hosts, 1.8 × 10^6^ CFU/mL). The 95% confidence interval for the relative risk crosses 1, so it was not statistically significant.

Next, the contribution of DNA polymerase IV to the frequency of nalidixic acid resistance was examined. A *dinB*-overexpression plasmid, pGEM-T:*dinB*, was constructed, and ghost attack experiments were performed. The number of mutator strains with W3110 pGEM-T:*dinB* was 7.0 × 10^7^ CFU/mL (7.0%) with significant relative risk (Table 1), which did not differ from that obtained with W3110 in the ghost attack experiment shown in Table 1. Mutator strains derived from W3110 and W3110 pGEM-T had 85- and 44-fold increases in mutant frequency compared with the parental strain, respectively (Figure 1E). In contrast, mutator strains derived from W3110 pGEM-T:*dinB* exhibited a 1,060-fold increase (a further 24-fold increase compared to the mutator derived from W3110 pGEM-T) in mutant frequency, although the mutant frequency of W3110 pGEM-T:*dinB* without attack by T4 ghosts (4.4 × 10^−9^) was similar to that of W3110 and W3110 pGEM-T. The mutant frequencies of all survived non-mutator strains were similar to those of the parent strains. Thus, higher mutant frequency of nalidixic acid resistance by DNA polymerase IV was observed only after an attack by T4 ghosts.

**Figure 2:**
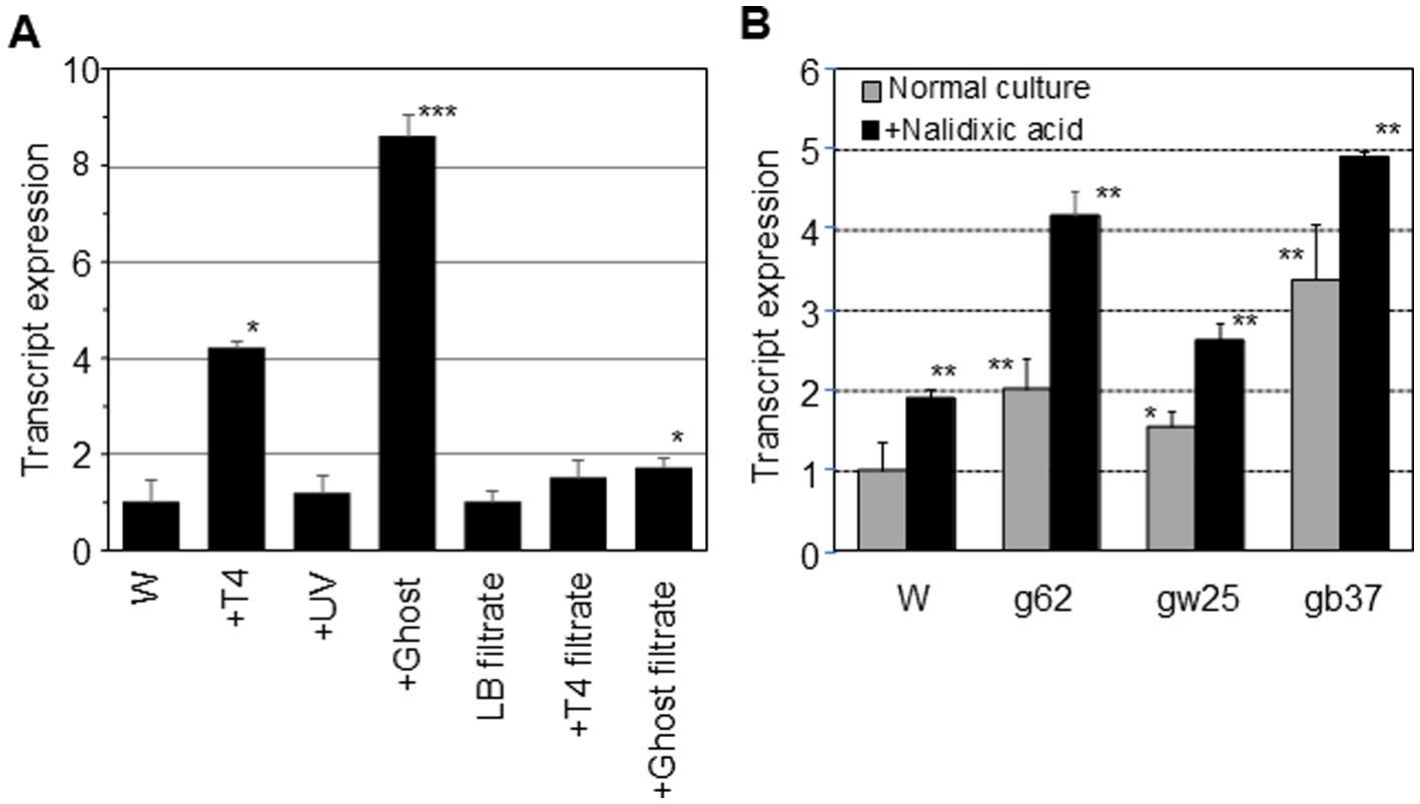
*dinB* mRNA expression in *E. coli*. qRT-PCR was performed with *E. coli* W3110 RNA as a template before and after incubation with intact phage T4 or UV-irradiated phage or ghost or particle-free supernatant derived from cells damaged by ghost attack **(A)**. The asterisk indicates statistical significance (*p < 0.05, *** *p* < 0.001) compared with the W3110 strain (W) treated under same conditions. qRT-PCR was also performed with mutator strains derived from ghost infection after incubation in LB broth with or without nalidixic acid **(B)**. The asterisk indicates statistical significance (*p < 0.05, ** *p* < 0.01) compared with the W3110 strain (W) in LB both without nalidixic acid.

The *dinB* mRNA expression in *E. coli* W3110 and ghost-derived mutator strains was examined by qRT-PCR. After incubation with the ghosts at a ratio of 1:1 for 5 min, the amount of *dinB* mRNA was increased by 8.6-fold compared to the W3110 parental strain (*p* < 0.001), which was incubated with the same buffer used for the ghost treatment but without the presence of ghosts (Figure 2A). The amount of *dinB* mRNA with ghosts was greater than that with intact or UV-irradiated phages.

The amount of *dinB* mRNA after exposing particle-free supernatant derived from cells damaged by ghost attack was slightly increased (1.7-fold) as compared with the W3110 parental strain suspended in LB filtrate (*p* < 0.05) but was much lower than that exposed to ghosts (Figure 2A). The adsorption of ghosts strongly induced DNA polymerase IV expression, but the induction effect of particle-free supernatant from ghost-infected cells was weak.

The mRNA expression from *dinB* in ghost-derived mutator strains was examined during the mid-log phase under normal culture conditions in LB broth by qRT-PCR. The amount of *dinB* mRNA was 1.5- to 3.4-fold higher than that in the W3110 parent strain (*p* < 0.05 for gw25 and *p* < 0.01 for gb37 and g62; Figure 2B). After incubation with nalidixic acid in LB broth for 5 min, the amount of *dinB* mRNA was further increased by 1.5- to 2.0-fold before incubation in all strains (*p* < 0.05; Figure 2B). Taking these results into account, attack by T4 ghosts might have stimulated the development of DNA polymerase IV-mediated nalidixic acid resistance in mutator strains.

### 3.4 Genetic characterization of mutator strains

To determine whether adsorption of T4 ghosts to host cells induced DNA substitution/insertion/deletion in the genome, whole-genome sequencing was performed with ghost-derived mutator strains. The phage T4 DNA was not detected in ghost-derived mutator strains. Sixteen single nucleotide polymorphism (SNP) loci were identified in the mutator strains (Table 2). Of these, four mutations were located in non-coding regions, seven synonymous substitutions were found in *pinR*, *pinQ*, *tufB*, *rhsA*, and *rhsB,* and five non-synonymous substitutions were detected in *flgG*, *tfaR*, *marR*, *frdD*, and *fbp*. The patterns of SNP loci varied by strain; notably, two non-synonymous substitutions, in *tfaR* and *marR*, were common among the mutator strains.

**Table 2:** Point mutations in mutator strain genomes.

Position ^a^	Strain						AA change	*gene*	Product	mRNA ^b^	Increase in mutant frequen^c^
	W3110	g25	g37	g62	g74	g94					
1,135,959	C	C	C	**T**	**T**	**T**	**Q164X**	***flgG***	**Flagellar basal-body rod protein**	**1.33**	**0.99** ^c^
1,433,535	G	**A**	**A**	**A**	**A**	**A**	**D83N**	***tfaR***	**Tail fiber assembly protein**	**0.65**	**78** ^d^
1,434,106	A	A	A	**T**	A	A	silent	*pinR*	Recombinase family protein	0.41	NA ^e^
1,434,250	A	A	**C**	A	A	A	silent	*pinR*	Recombinase family protein	0.41	NA
1,622,855	T	**G**	**G**	**G**	**G**	**G**	**X131E**	***marR***	**DNA-binding transcriptional repressor**	**0.21**	**45** ^d^
1,637,269	T	T	**G**	T	T	T	silent	*pinQ*	Recombinase family protein	0.22	NA
1,637,413	T	T	**A**	T	T	T	silent	*pinQ*	Recombinase family protein	0.22	NA
1,982,985	-	-	-	-	**G**	-	NA	*flhD/insH1*	Flagellar transcriptional regulator /IS5 family transposase	NA	NA
3,466,361	A	**G**	**G**	**G**	A	**G**	silent	*tufB*	Elongation factor Tu	1.25	NA
3,700,840	T	**C**	T	T	T	T	NA	*gltU/rrsC*	tRNA-Glu/16S ribosomal RNA	NA	NA
3,881,360	T	T	T	**C**	T	**C**	silent	*rhsA*	RHS element protein	1.10	NA
4,024,351	T	T	**C**	T	T	T	silent	*rhsB*	RHS element protein	0.92	NA
4,388,544	G	**A**	G	G	G	G	**T118I**	***frdD***	**fumarate reductase subunit**	**1.30**	**0.95** ^**c**^
4,464,811	T	T	**G**	T	T	T	**D110A**	***fbp***	**Class 1 fructose-bisphosphatase**	**1.40**	**3.3** ^c^
4,552,856	C	-	C	C	C	-	NA	pseudogene	pseudogene (tyrosine recombinase)	NA	NA
4,552,999	A	A	**G**	A	A	**G**	NA	pseudogene/*fimA*	pseudogene/type 1 fimbrial major subunit	NA	NA

a Position in *E. coli* str. K-12 substr. W3110.

b Relative mRNA level of the corresponding gene in ghost-derived mutator strains was compared with it in the parent strain.

c Median mutant frequency of nalidixic acid resistance in the parent strain carrying mutant gene on plasmid was compared with that in the parent strain carrying wild type gene on plasmid.

d Median mutant frequency of nalidixic acid resistance in gene-deficient strain was compared with that in the parent strain.

e Not Applicable.Bold values indicate non-synonymous substitution.

TfaR is a phage tail fiber assembly protein on prophage regions. MarR is a DNA-binding transcriptional repressor of the *marRAB* operon and negatively autoregulates its own expression. MarA is a global transcriptional regulator of >100 chromosomal genes, such as resistance to antibiotics, oxidative stress, and organic solvents (Ariza et al., 1994; Pomposiello et al., 2001). The microarray analysis showed that the expression level of the *stfR*-*tfaR*, and *marRAB* operon in ghost-derived mutator strains decreased by 0.44-fold (0.65 for *tfaR*, *p* < 0.001) and 0.20-fold (0.21-fold for *marR*, *p* < 0.001), respectively.

The functions of these genes are not related directly to DNA repair, and thus the contribution of these non-synonymous substitutions to the frequency of nalidixic acid resistance was examined. Since the mRNA expression level of the *marR* and *tfaR* mRNA had decreased, these gene-deficient strains were constructed. The *marR*-deficient and *tfaR*-deficient strains showed that mutant frequencies were 45-fold and 78-fold higher than that in the W3110 parental strain, respectively (Table 2).

On the other hand, given the observed upregulation of *flgG, frdD*, and *fbp*, mutant gene-overexpression plasmids were constructed and introduced into the *E. coli* W3110 parental strain to assess their impact. Non-synonymous substitutions on *flgG*, *frdD*, and *fbp* contributed only marginally to the frequency of nalidixic acid resistance or no effect. BLAST search showed that these mutants on the *marR* and *tfaR* were found in many *E. coli* strains deposited in GenBank. Although the mutant frequencies of the strains in GenBank are unclear, it can be said that these mutations could occur in nature.

### 3.5 Phage resistance and stress resistance of mutator strains

#### 3.5.1 Phage resistance

**Figure 3:**
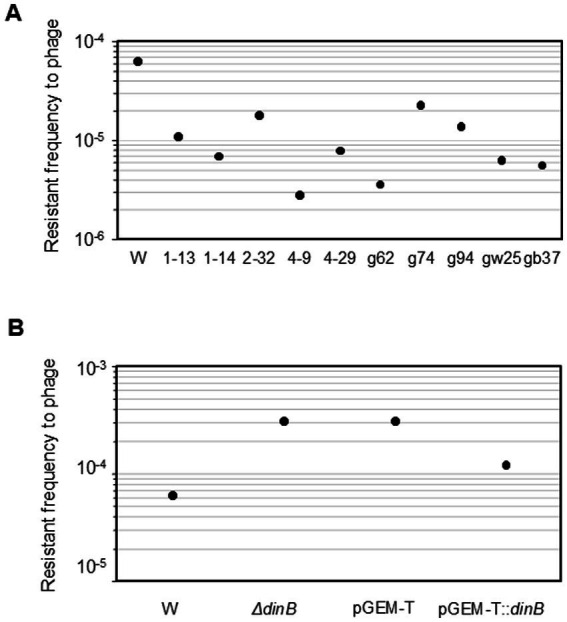
Frequency of resistance to phage T4 and role of DNA polymerase IV in phage resistance. The frequency of resistance to phage T4 in mutator strains **(A)** in *dinB*-deficient mutant, *dinB*-overexpression strain **(B)** was shown as median of 10 independent cultures.

The frequency of resistance to phage T4 was examined (Figure 3A). All phage-derived mutator strains (1–13, 1–14, 2–32, 4–9, and 4–29) exhibited a 0.04- to 0.17-fold lower frequency of resistance than the W3110 parental strain (*p* < 0.01). Ghost-derived mutator strains (g62, gw25, and gb37) showed 0.06- to 0.37-fold lower frequency of resistance to phage T4 (*p* < 0.05 for gw25 and *p* < 0.01 for others) compared to the W3110 parental strain.

The phage adsorption assay showed that phage T4 adsorbed to ghost-derived mutator strains more efficiently than to the W3110 parental strain (*p* < 0.01 for gw25 at 5 min and g62 for 10 min, *p* > 0.05 for gb37 for 1 min, and *p* < 0.05 for others; Supplementary Figure 1). The effective adsorption might contribute to the lower frequency of phage resistance.

The contribution of DNA polymerase IV to the frequency of phage resistance was also examined (Figure 3B). In the *dinB*-deficient strain, the frequency of phage resistance increased by 4.9-fold (*p* < 0.01). The W3110 pGEM-T:*dinB* had a 0.39-fold lower frequency than the W3110 pGEM-T strain (*p* < 0.01). Therefore, DNA polymerase IV might be involved in the reduction in the frequency of phage resistance. Taking into account these results, the attack by T4 phage ghosts might have introduced mutations and allowed DNA polymerase IV to be expressed more readily, thereby promoting the development of DNA polymerase IV-mediated nalidixic acid resistance and reduction in phage resistance in escaped host cells.

#### 3.5.2 Host cell survival (stress resistance)

**Figure 4:**
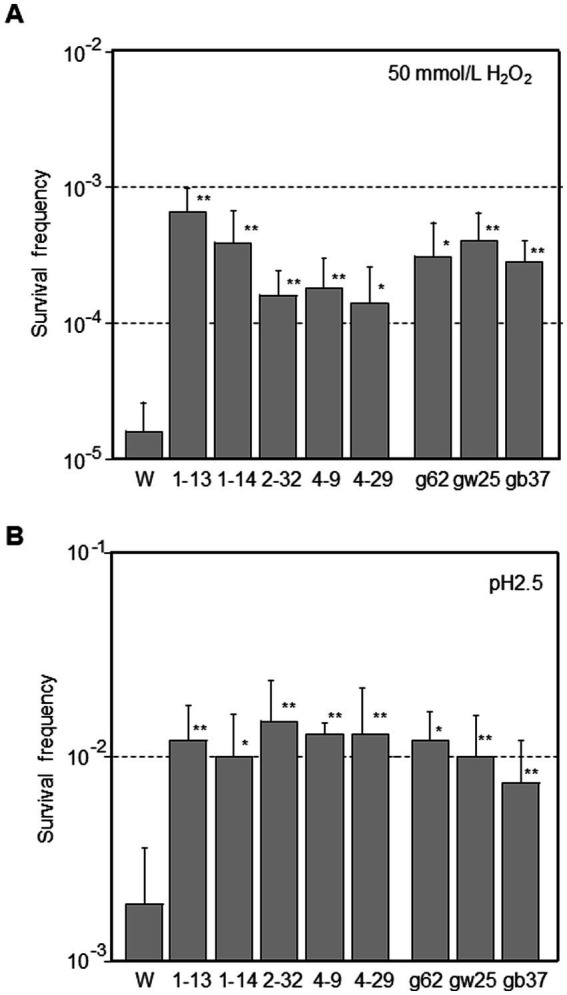
Determination of resistance levels against stresses in mutator strains derived from phage or ghost infection. *E. coli* were grown to the mid-log phase in LB broth (pH 7.0). Cells were diluted into LB broth containing 50 mmol/L H_2_O_2_ **(A)** or M9 minimal medium supplemented with 0.4% glucose and 1.5 mmol/L glutamate **(B)** and incubated for 15 min at 37 °C. The initial cell densities were about 10^8^ CFU/mL. Survival frequency was defined as the ratio of CFU on plates after exposure to the number of CFU on plates before exposure. Data are the means from five independent experiments. Error bars represent standard deviations of the mean. The asterisk indicates statistical significance (^*^p < 0.05, ^**^p < 0.01) compared with W3110 parental strain (W) treated under same conditions.

Stress resistance was examined in mutator strains. *E. coli* cells often encounter severe changes in pH throughout the gastrointestinal tract and oxidative stress in various environments. ROS are produced by a variety of compounds and enzymes in these environments, which can damage DNA, RNA, proteins, and lipids. Phage-derived mutator strains had 8.7- to 42-fold higher survival frequencies after exposure to 50 mmol/L H_2_O_2_ than the W3110 parental strain (*p* < 0.05 for 4–29 and *p* < 0.01 for others; Figure 4A). Ghost-derived mutator strains showed an 18- to 25-fold higher frequency of resistance to oxidative stress (*p* < 0.05 for g62 and *p* < 0.01 for others).

*E. coli* has developed at least four Acid Resistance (AR) systems. The glutamate-dependent AR2 system plays a major role in acid resistance (Lu et al., 2013). Thus, a bacterial survival assay was employed to investigate AR2 (Figure 4B). Mutator strains had 3.3- to 5.0-fold higher survival frequencies than the W3110 parental strain after exposure to pH 2.5 (*p* < 0.05 for 1–14 and *p* < 0.01 for others). Ghost-derived mutator strains showed 3.9- to 4.4-fold higher frequency of resistance to acid stress (*p* < 0.05 for g62 and *p* < 0.01 for others). Consequently, mutator strains were more sensitive to phage attack but more resistant to oxidative and acid stresses than the W3110 parental strain. These characteristics of mutator strains might benefit progeny phages in future phage attacks.

#### 3.5.3 Fitness

Competition experiments were conducted to examine whether the ghost-derived mutator conferred a relative fitness benefit compared to the parental strain. The average competitive indexes (ghost-derived mutator strain to parental strain ratio) were 4.00 in g62, 2.76 in gw25, and 2.18 in gb37. The fitness advantage of these ghost-derived mutator strains might have contributed to the survival of mutator strains among the wild-type population. Ghost adsorption might increase the phage sensitivity of host cells that escaped lysis and promote their survival to increase the probability of producing progeny virions in future phage attacks.

#### 3.5.4 *gyrA* mutation

To verify that the resistance was associated with a chromosomal mutation, the sequences on *gyrA* in Nal^R^ mutants derived from mutator strains were compared to Nal^R^ mutants derived from the parent strain (Supplementary Table 2). The base-substitution mutation in mutator strains was dominated by A: T to C: G (>640-fold) transversion, G: C to T: A (103-fold) transversion, and G: C to A: T (77-fold) transition. These base-substitution mutation patterns were clearly different from those in the parent strain.

### 3.6 Transcriptome characterization of ghost-derived mutator strains

To understand the characteristics of mutator strains at the mRNA level, a global transcriptome analysis of ghost-derived mutator strains using microarrays was conducted. Upregulated and downregulated operons during the mid-log phase were compared with the W3110 parental strain.

#### 3.6.1 Phage sensitivity

**Figure 5:**
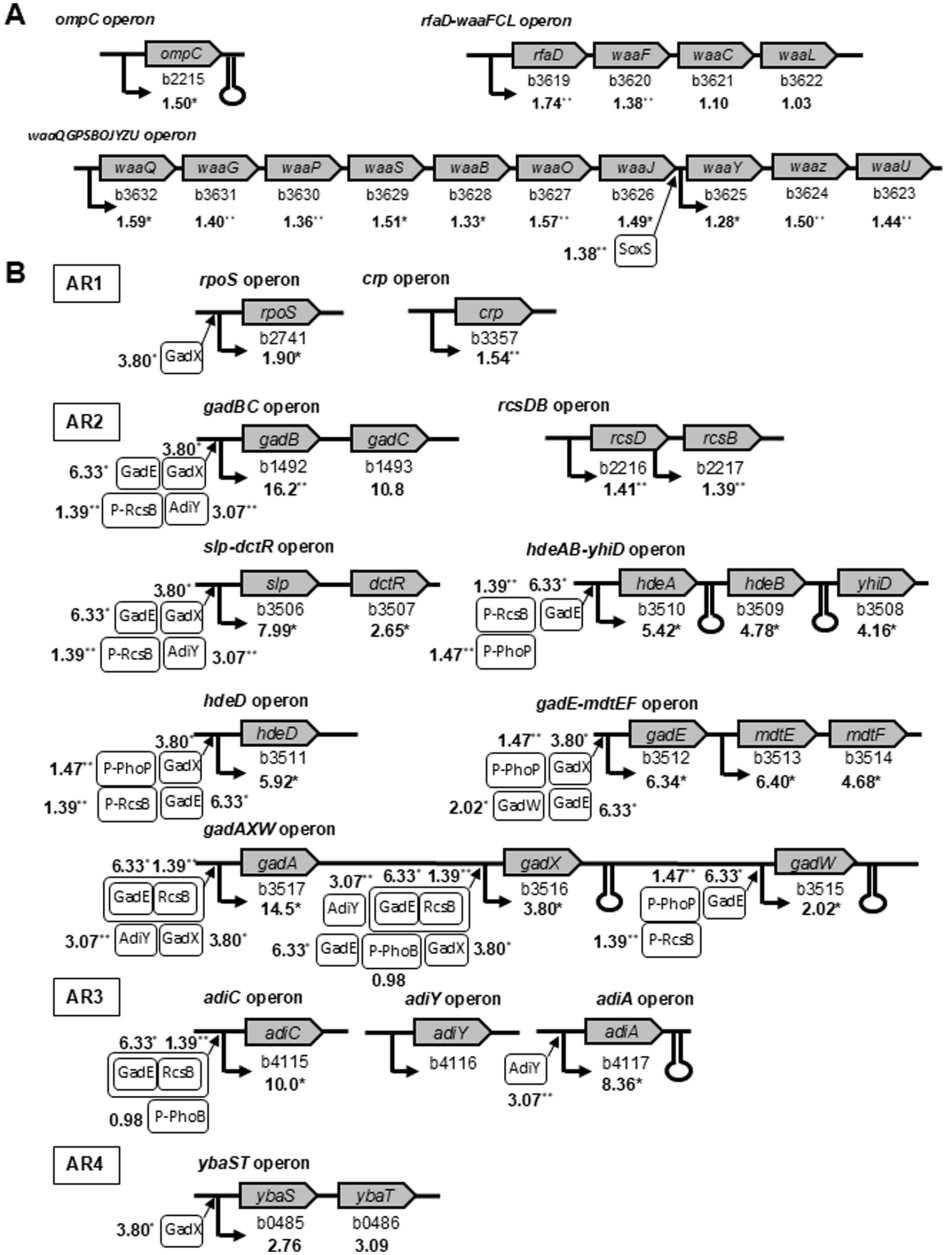
Illustration of selected *E. coli* operons identified by microarray analysis to be upregulated in ghost-derived mutator strains. Operons were involved in phage receptor **(A)** and acid resistance **(B)**. The average mRNA level ratios in three ghost-derived mutator strains are given under each gene. The asterisk indicates statistical significance (^*^p < 0.05, ^**^ *p* < 0.01) compared with the W3110 parental strain treated under same conditions. The regulation summary diagrams are referred to EcoCyC (https://biocyc.org/). The known promoters are marked by bold arrows. Terminators are shown as hairpins. Boxed gene products with arrows simulate the activity of the given gene products on the operon.

A combination of lipopolysaccharide (LPS) and an outer membrane protein (OmpC) served as the receptor for phage T4 (Henning and Jann, 1979). The genomic loci (b3619–b3632) in the *rfaD-waaFCL* and *waaQGPSBOJYZU* operons are involved in LPS biosynthesis. These operons and the *ompC* operon genes were upregulated in the ghost-derived mutator strains (Figure 5A). The expression of *soxS*, which induces *waaY-waaZ* in the middle of the LPS core biosynthetic gene cluster (Lee et al., 2009), increased by 1.38-fold (*p* < 0.01). Previous studies on LPS and OmpC mutants have shown that the absence of at least one receptor decreases infection efficiency, whereas the loss of both receptors leads to phage resistance (Yu and Mizushima, 1982). The higher amounts of mRNA related to LPS biosynthesis and OmpC genes at the operon level supported the reduced frequencies of resistance to phage T4 and increased adsorption to the cell receptors in mutator strains (Figure 3).

#### 3.6.2 Host cell survival

The cellular concentrations of many proteins were elevated above their basal levels in response to the increased flux of H_2_O_2_ and other organic peroxides (Farr and Kogoma, 1991). The upregulated genes known to be involved in defense against oxidative stress in ghost-derived mutator strains are shown in Table 3. In particular, *soxS* and OxyR-regulated genes involved in defense against oxidative stress exhibited slight but significant increases in their mRNA levels.

**Table 3:** Relative mRNA levels for upregulated genes involved with defense against oxidative stress in three ghost-derived mutator strains.

Gene name	Designation	Protein function	mRNA^a^	Major known activator
*ksgA*	b0051	16S rRNA methyltransferase	1.57^**^	Fis
*ahpF*	b0606	Alkyl hydroperoxide reductase	1.53^*^	OxyR
*ahpC*	b0605	Alkyl hydroperoxide reductase	1.52^*^	OxyR
*gor*	b3500	Glutathione oxidoreductase	1.49^**^	OxyR
*polA*	b3863	DNA polymerase I	1.48^**^	DnaA
*oxyR*	b3961	Oxidative stress transcriptional regulator	1.41^**^	cAMP-Crp/Hydrogen peroxide
*soxS*	b4062	Regulation of superoxide response	1.38^**^	SoxR, AcrR, SoxS
*katG*	b3942	Catalase; hydroperoxidase HPI	1.37^**^	OxyR

a Relative mRNA level was shown as average of those in three ghost-derived mutator strains.

* *p* < 0.05, ** *p* < 0.01.

Many genes in different operons are involved in AR systems (Lu et al., 2013; Masuda and Church, 2003; Richard and Foster, 2004; Bak et al., 2014). Some features related to *rpoS* and *crp* are known in AR1, but the full set of components has yet to be identified. In the glutamate-dependent AR2 system, genomic loci (b3506–b3517) in *slp-dctR*, *hdeAB-yhiD*, *hdeD*, *gadE-mdtEF*, and *gadAXW* operons function with *gadBC* and *rcsDB* operons as regulators. *adiC*, *adiY*, and *adiA* operons are involved in the arginine-dependent AR3 system. The *ybaS*-*gadC* system works together with *gadA*/*gadB* gene products in L-glutamine-dependent AR4 (Lu et al., 2013). This microarray analysis showed that these operons involved in these AR systems were upregulated in ghost-derived mutator strains (Figure 5B). In addition, the major genes involved in the positive regulation of the AR system, such as *phoB*, *phoP*, and AR gene products (*gadE*, *gadX*, *gadW*, and *adiY*), were also upregulated.

The changes in mRNA levels of the genes described above are likely to contribute to the different phenotypic characteristics (phage sensitivity, acid resistance, and oxidative stress resistance) of ghost-derived mutator strains. Quantitative reverse transcription-polymerase chain reaction (qRT-PCR) was carried out on seven genes (*ompC, rfaD, gadB, slp, gadE, ksgA,* and *ahpF*) *to* validate the DNA microarray analysis (Supplementary Figure 2). These results indicated that all genes in the three ghost-derived mutator strains (g62, gw25, and gb37; Table 3; Figure 5) were significantly upregulated (*p* < 0.05) in accordance with the DNA microarray results.

### 3.7 Effect of ghost adsorption on the gene expression profile of the host

To examine whether ghost adsorption directly affects the expression of genes involved in phage sensitivity and host cell survival (stress resistance), a global transcriptome analysis was performed using microarrays with host cells 10 min after the ghost attack.

Eighty genes on 50 operons had significantly increased mRNA levels at the operon level after attack by the ghosts (*p* < 0.05 at the average operon level; Supplementary Table 3). The main upregulated genes belonged to operons encoding proteins involved in the metabolism and transport of phosphate, metabolism and transport of nitrogen, biosynthesis and transport of arginine, transport and phosphorylation of fructose, and magnesium transport (Table 4). There were significant decreases in the mRNA levels of 133 genes on 77 operons at the operon level after attack by the ghost phages (*p* < 0.05 at the average operon level; Supplementary Table 4). The downregulated operons were involved in the transport of sugar, sugar alcohol, glycerol, amino acids, and other molecules. Interestingly, the most highly upregulated genes involved in acid resistance and defense against oxidative stress and phage sensitivity in ghost-derived mutator strains exhibited no significant changes after ghost attack. However, as shown in Table 4, *phoB-phoR* had a 20-fold increase in expression, whereas *soxS* expression increased by 2.56-fold (*p* = 0.10).

**Table 4:** Relative mRNA levels for upregulated operons in ghost infected *E. coli* W3110.

Gene		Protein function	mRNA^a^	*Z* score	*p*-value	Ratio to phage^b^
Designation	Name					
Phosphate metabolism, transport and the regulon						
b0241	*phoE*	outer membrane pore protein E	6.53	3.30	0.001	1.27
b0383	*phoA*	alkaline phosphatase	6.08	3.17	0.002	1.21
b0384	*psiF*	induced by phosphate starvation	9.30	3.94	0.000	1.28
b0399	*phoB*	positive response regulator for pho regulon, sensor is PhoR	21.69	5.45	0.000	5.23
b0400	*phoR*	positive and negative sensor protein for pho regulon	18.37	5.16	0.000	5.15
b1020	*phoH*	PhoB-dependent, ATP-binding pho regulon component; may be helicase; induced by P starvation	3.45	2.16	0.031	1.17
b3724	*phoU*	negative regulator for pho regulon and putative enzyme in phosphate metabolism	11.72	4.35	0.000	5.13
b3725	*pstB*	ATP-binding component of high-affinity phosphate-specific transport system	14.51	4.73	0.000	6.29
b3726	*pstA*	high-affinity phosphate-specific transport system	16.24	4.94	0.000	6.61
b3727	*pstC*	high-affinity phosphate-specific transport system, cytoplasmic membrane component	19.17	5.23	0.000	7.89
b3728	*pstS*	high-affinity phosphate-specific transport system; periplasmic phosphate-binding protein	32.68	6.19	0.000	7.58
b4030	*psiE*	phosphate starvation-inducible protein	3.53	2.20	0.028	0.64
Nitrogen metabolism, transport and the regulon						
b1221	*narL*	pleiotrophic regulation of anaerobic respiration: response regulator for nar, frd, dms and tor genes	4.55	2.66	0.008	2.99
b1222	*narX*	nitrate/nitrate sensor, histidine protein kinase acts on NarL regulator	3.90	2.38	0.017	2.66
b1223	*narK*	nitrite extrusion protein	14.46	4.73	0.000	7.52
b1224	*narG*	nitrate reductase 1, alpha subunit	12.04	4.40	0.000	3.91
b1225	*narH*	nitrate reductase 1, beta subunit	12.72	4.50	0.000	3.40
b1226	*narJ*	nitrate reductase 1, delta subunit, assembly function	9.05	3.89	0.000	2.36
b1227	*narI*	nitrate reductase 1, cytochrome b	1.79	0.99	0.324	1.01
b3365	*nirB*	nitrite reductase	11.05	4.25	0.000	2.90
b3366	*nirD*	nitrite reductase	12.10	4.41	0.000	2.63
b3367	*nirC*	nitrite reductase activity	6.73	3.36	0.001	3.00
b3368	*cysG*	uroporphyrinogen III methylase; sirohaeme biosynthesis	2.96	1.89	0.059	2.88
Arginine biosynthesis and transport						
b0032	*carA*	carbamoyl-phosphate synthetase, glutamine	3.20	2.03	0.043	4.03
b0033	*carB*	carbamoyl-phosphate synthase large subunit	3.29	2.08	0.038	4.70
b0273	*argF*	ornithine carbamoyltransferase 2, chain F	7.68	3.59	0.000	4.82
b0860	*artJ*	arginine 3rd transport system periplasmic binding protein	9.31	3.94	0.000	7.00
b0861	*artM*	arginine 3rd transport system permease protein	4.16	2.49	0.013	9.98
b0862	*artQ*	arginine 3rd transport system permease protein	4.07	2.46	0.014	9.33
b0863	*artI*	arginine 3rd transport system periplasmic binding protein	4.96	2.81	0.005	8.70
b0864	*artP*	ATP-binding component of 3rd arginine transport system	4.45	2.62	0.009	9.92
b2818	*argA*	N-acetylglutamate synthase; amino acid acetyltransferase	4.80	2.75	0.006	3.19
b3172	*argG*	argininosuccinate synthetase	4.37	2.58	0.010	5.13
b3958	*argC*	N-acetyl-gamma-glutamylphosphate reductase	12.79	4.51	0.000	7.14
b3959	*argB*	acetylglutamate kinase	5.88	3.12	0.002	6.77
b3960	*argH*	argininosuccinate lyase	6.67	3.34	0.001	7.06
b4254	*argI*	ornithine carbamoyltransferase 1	7.92	3.65	0.000	4.92
Fructose transport and phosphorylation						
b2167	*fruA*	PTS system, fructose-specific transport protein	2.13	1.30	0.194	0.38
b2168	*fruK*	fructose-1-phosphate kinase	3.27	2.07	0.039	0.33
b2169	*fruB*	PTS system, fructose-specific IIA/fpr component	4.25	2.53	0.011	0.39
Magnesium transport						
b4242	*mgtA*	Mg2 + transport ATPase, P-type 1	4.51	2.64	0.008	0.44

a Relative mRNA level in ghost infected *E. coli* W3110 cells was compared with it when addition of SM buffer, and shown as average of those in three independent experiments.

b Ratio of relative mRNA levels in ghost infected cells to those in phage infected cells.

The *dinB* gene is regulated by the LexA DNA-binding transcriptional repressor, RpoS, and Mg^2+^ (Wagner et al., 1999; Galhardo et al., 2009; Storvik and Foster, 2010). Microarray analysis showed that the expression level of the *lexA-dinF* operon decreased by 0.75-fold (*p* = 0.55) and that of the Mg^2+^ transport ATPase gene (*mgtA*) and *rpoS* increased by 4.5-fold (*p* = 0.008) and 2.1-fold (*p* = 0.34), respectively.

In contrast to phage attack, about 70 to 80% of host cells remained viable after ghost attack (Table 1). That is, host cells were exposed to stresses via material transport after ghost adsorption, but most of them remained viable. Ghost adsorption caused a change in the expression of hundreds of genes via the transport of material across the cell membrane. The *marR* and *tfaR* mutations and the changes in gene expression levels of global transcription regulators might have triggered the expression of genes associated with phage receptors, acid resistance, and oxidative stress resistance.

## 4 Discussion

In this study we showed that adsorption of the phage to the host cell surface changed the mutant frequency of host cells that escaped phage-induced lysis (Figure 1; Table 1), changed the phage sensitivity to make lysis easier in future phage attacks (Figure 3), and promoted the survival of escaped host cells under stress conditions (acid and ROS) (Figure 4). Thus, the possible mechanisms related to the emergence of mutator strains in the population were investigated.

It is important to determine whether the emergence of mutator strains was due to phage exposure or the preexistence of mutator strains before phage exposure. It is possible that preexisting mutator strains and phage receptor-less mutants could be closely related and that phage T4 (or the ghost) could have selected phage receptor-less mutants. Alternatively, the preexisting mutator strains and phage receptor-less mutants might not be related, and phage T4 (or the ghost) could have killed phage-sensitive cells. In this case, the preexisting mutator strains should have become more readily detectable in lines exposed to phages than in those not exposed to them. To rule out these possibilities, this study investigated the possible emergence of mutator strains owing to phage exposure. If an interaction occurred between the bacteria and phages on the agar plate, the receptor-less mutants should have grown better after the phage attack. However, phage resistance tests showed that all phage- or ghost-derived mutator strains were sensitive to phage T4 (Figure 1A). Therefore, the emergence of mutator strains was not due to the selection of phage receptor-less mutants after phage (or ghost) attack. If mutator strains preexisted in the W3110 population, they should have emerged at a rate of 1.2 × 10^7^ CFU/mL (1.2% of the initial host). However, no colony in the W3110 population without phage exposure had a median mutant frequency above 10^−7^ (<1.1 × 10^6^ CFU/mL, Table 1). Relative risk and the 95% confidence interval supported the hypothesis that the emergence of mutator strains was due to phage (ghost) exposure.

### 4.1 Molecular mechanisms

The response of host cells after ghost adsorption is summarized in Supplementary Figure 3. The molecular mechanisms behind the change in the phenotype of escaped cells after the ghost attack still need to be identified in future studies. The emergence of mutator strains following phage ghost attack suggests that adsorption of phage ghosts acts as a trigger for the mutant with elevated mutation frequencies (Table 1). If the entry of foreign DNA was responsible for the emergence of mutators, it would be expected that exposure to ghosts, which lack nucleic acids, would reduce their incidence. Contrary to this expectation, our findings demonstrated a promoting effect, indicating that the emergence of mutator strains is not attributable to the influence of foreign DNA. Additionally, supernatant exposure experiments showed that the increased frequency of mutator strains was not caused by extracellular substances released from damaged cells, further supporting the conclusion that ghost adsorption itself is a key factor in mutator emergence.

Adsorption of ghosts to the host cell surface induced changes in material transport across cell membranes and led to alterations in global transcription profile (Table 4; Supplementary Tables 3, 4). Notably, the mRNA levels of DNA polymerase IV were markedly increased (Figure 2A), resulting in DNA polymerase IV-mediated mutations in the host genome, such as those in *tfaR* and *marR* (Table 2). Results about suppression of emergence of mutator in the *dinB*-deficient strain would support this notion (Table 1). These mutations, along with corresponding changes in mRNA expression, subsequently triggered the activation of genes associated with phage receptors, acid resistance, and oxidative stress resistance in mutator strains.

#### 4.1.1 Higher mutant frequency

The high mutant frequency in mutator strains was associated with higher DNA polymerase IV activity (Figures 1E, 2B; Table 1). MarA has been known to indirectly reduce RpoS which is one of *dinB* regulators (Ruiz et al., 2008). Since MarR functions as a negative autoregulator of its own expression within the *marRAB* operon (Alekshun et al., 2000; Pomposiello et al., 2001), *marR* mutation and the lower mRNA expression might have caused the higher expression level of *dinB* mRNA, thereby promoting the development of DNA polymerase IV-mediated mutation in host cells that escaped lysis (Figure 1E). Although transcription of *tfaR* was induced upon biofilm formation, its function in *E. coli* remains unknown (Ren et al., 2004). In the present study, we demonstrated that *tfaR* contributed to the increased frequency of mutator phenotypes (Table 2). However, the relationship between *tfaR* and *dinB* remains to be elucidated in future investigations.

DNA polymerase IV is a mutation-promoting enzyme specifically required for most adaptive point mutations (Mckenzie et al., 2001). Its overproduction causes hypermutation, including −1 frameshifts and some substitutions (Wagner et al., 1999; Galhardo et al., 2009). One important function of DNA polymerase IV in *E. coli* in adaptive point mutation involves environmentally inducible genetic changes. A previous study showed that DNA polymerase IV expression specifically enhances changes in A:T to C:G, A:T to G:C, and C:G to G:C (Wagner and Nohmi, 2000). The *gyrA* sequencing in the Nal^R^ mutant in this study showed that the development of nalidixic acid resistance in mutator strains resulted from mutation of the *gyrA* sequence, especially transversion (Supplementary Table 2). If DNA polymerase IV activity contributed to *gyrA* mutation, these results were consistent with the previous report.

#### 4.1.2 Higher frequency of resistance to oxidative stress

Adsorption of phage ghosts has been shown to have various effects on cells, such as inhibition of DNA/RNA/protein synthesis or enzyme induction, release of cations and metabolites from cells, depolarization of the cytoplasmic membrane, and death (Duckworth, 1970; Shapira et al., 1974; Kuhn and Kellenberger, 1985). Phage ghost adsorption also affects the uptake and release of inorganic and organic phosphates. Ghosts of phage T4 inhibit the uptake of glucose-6-phosphate and cause loss of ATP from host cells, and the intracellular level of ATP is decreased in ghost-infected cells (Winkler and Duckworth, 1971). The present study demonstrated that ghost adsorption induced changes in the expression of genes associated with various material transport and metabolic processes, as well as global transcriptional regulators, including the PhoB-PhoR system (Table 4).

The two-component regulatory system PhoB-PhoR senses the environmental phosphate concentration and responds to phosphate starvation. PhoR (sensor kinase) senses when the concentration of environmental phosphate is low and activates PhoB (response regulator and transcriptional activator) by phosphorylation (VanBogelen et al., 1996). Glucose metabolism in phosphate-starved *E. coli* leads to an increase in oxidative damage. The function of PhoB is related to sensitivity to H_2_O_2_ (Moreau, 2004; Yuan et al., 2005)_._ Membrane perturbation, cell wall damage, or inhibition of essential cytoplasmic activities by diffusible molecules induces ROS production (Dong et al., 2015). The change in intracellular phosphate concentration in host cells after adsorption of ghosts might trigger the upregulation of OxyR- or SoxR-regulated genes in the mutator strains.

#### 4.1.3 Lower frequency of resistance to phage

The DNA-binding transcriptional repressor AcrR contains a binding site for the homologous transcriptional activator MarA in the promoter region (Martin et al., 1999). AcrR represses the transcription of *soxS*, which is involved in LPS biosynthesis and regulation of superoxide response (Lee et al., 2009; Lee et al., 2014). The expression of *acrR* showed a 0.61-fold decrease, whereas *soxS* expression increased (Figure 5; Table 3). Therefore, the decrease in *marA* and *acrR* mRNA could lead to higher *soxS* expression in ghost-derived mutator strains than in the parent strain. SoxS induces *waaY*-*waaZ* in the middle of the LPS core biosynthetic gene cluster (Lee et al., 2009). In addition, DNA polymerase IV was shown to be involved in reducing the frequency of phage resistance (Figure 3). The activity of DNA polymerase IV might have been affected by MarA and RpoS as discussed above. Therefore, the mutation in *marR* and lower mRNA expression might have contributed to the higher frequency of resistance to oxidative stress and lower frequency of resistance to phage.

#### 4.1.4 Higher frequency of resistance to acid

Many genes involved in AR are activated by phosphorylated PhoB or PhoP or by their activated gene products, such as GadX and GadW (Figure 5B). MarA indirectly reduced the activity of regulators GadX and GadW (Ruiz et al., 2008). Higher *gadX* and *gadW* expression was found in ghost-derived mutator strains. Therefore, the *marR* mutation and lower mRNA expression might have also contributed to the higher frequency of resistance to acid stress.

### 4.2 Biological and ecological significance

All mutator strains showed a higher frequency of resistance to acid and oxidative stresses but a lower frequency of resistance to phages than the W3110 parental strain. Elevation in the frequency of resistance to acid and oxidative stress and reduction in phage resistance might be related to the mutations and their mRNA expression through regulation of GadX, GadW, RpoS, and SoxS. These phenotypic characteristics of mutator strains would be beneficial to progeny phages in future phage attacks. Therefore, it is suggested that ghosts have important effects on progeny phages in future phage attacks. Future studies should include a more detailed identification of multicomponent pathways involved in these mutator phenotypes.

Adsorption of phage ghosts to hosts is common among bacteria because phages outnumber bacteria by 10-fold in any given environment, and phage ghosts are produced naturally during propagation in host cells. A previous study showed that mutator strains of enteric bacteria occur in the environment at a frequency of approximately 1% (LeClerc et al., 1996). These results suggested that phage ghosts are one of the drivers that generate mutator strains in the environment. Gene transfer by phages in natural aquatic environments has been shown to occur at high frequencies in a diverse range of bacteria (Kenzaka et al., 2007; Kenzaka et al., 2010). Our results also suggested that a number of phage ghosts adsorbed to hosts affects prokaryotic diversity as well as phage-mediated gene transfer. In nature, the adsorption of functional virions is much greater than that of phage ghosts. The effect of sensitization against phages might be caused by the simple adsorption of the phage to the host cell surface. Future studies are required to study the effects of adsorption of intact phages that do not cause host death.

## Data Availability

The datasets presented in this study are publicly available. This data can be found at: https://www.ncbi.nlm.nih.gov/genbank, accession numbers CP084893-CP084899.

## Footnotes

^1^ http://www.shigen.nig.ac.jp/

^2^ http://www.ncbi.nlm.nih.gov/
