# Supplementary material for: Stress-resistant but phage-sensitive host mutants induced by phage T4 ghost adsorption
Source: Front Microbiol. 2025 Oct 10;16:1683709. doi: 10.3389/fmicb.2025.1683709 (PMC12549603; doi:10.3389/fmicb.2025.1683709)
Supplement: Supplementary file 1 [file Data_Sheet_1.PDF]

Supplementary Material

1 Supplementary Methods

Gene deletion strain. For the construction of the gene- deficient mutant strain, the Quick & Easy *E. coli* Gene Deletion Kit (Gene Bridges GmbH, Heidelberg, Germany) was employed according to the protocol recommended by the technical manual. *E. coli* W3110 was transformed with plasmid pRedET (Gene Bridges GmbH), and subsequently, the clones of interest were selected on LB agar plates containing 12.8 µg mL^{-1} tetracycline. In order to inactivate the active sites on the target gene by the insertion of FRT sites, the target sequence was selected by referring to the EcoCyc database (http://biocyc.org/). A linear DNA fragment FRT-flanked PGK-gb2-neo cassette flanked by 50-mer homologous sequences for target gene was generated by PCR with the following primer sets. dinB_upperOligo [5'- gtatactttaccagtgttgagaggtgagcaatgcgtaaaatcattcatgt*aattaaccctcactaaagggcg*-3'] and dinB_lowerOligo[5'-gggcgggattgtcgcgcatctccactgcggcgaaaaagcagtccatatcc*taatacgactcactatagggctc*-3']) for dinB. marR_upperOligo [5'-atggttaatcagaagaaagatcgcctgcttaacgagtatctgtctccgct*aattaaccctcactaaagggcg*-3'] and marR_lowerOligo[5'-ccgcgcagcggatagagcagagcaccttaaactgtgccgcggtaatatcc*taatacgactcactatagggctc*-3']) for marR, and combination of tfaR_upperOligo1 [5'-attccgc ctcatacagg tctgccagca aacagta *aattaaccctcactaaagggcg*-3'] and tfaR lowerOligo1 [5'-gttgaaaacagccacgaagc cagccggaatatctggcggtgcaatatcgg*taatacgactcactatagggctc*-3'] for 1st PCR and combination of tfaR_upperOligo2 [5'-tga aggtgatgca tatattccgc ctcatacagg tctgc-3'] and tfaR lowerOligo2 [5'-gttgaaaacagccacgaagc cagccgg-3'] for 2nd PCR for tfaR, were used (FRt- PgK-gb2-neo cassette sequence was given in italics).

The *E. coli* W3110 carrying the plasmid pRedET, in which the expression of the Red/ET recombination proteins was induced, was transformed with the above linear DNA fragment, comprising the FRT-flanked PGK-gb2-neo cassette. The FRT-flanked PGK-gb2-neo cassette was expected to recombine on the target gene. Subsequently, the clones of interest were selected on LB agar plates containing 25 µg mL^{-1} kanamycin after incubation at 37°C for 24 h. The plasmid pRedET disappeared during the incubation at 37°C. As the kanamycin selection marker was flanked by FRT sites, the cassette was removed by the transformation of the plasmid 707-FLPe (Gene Bridges GmbH) as described below: The transformants of *E. coli* W3110 with FRT sites and kanamycin selection marker on the chromosome and the plasmid 707-FLPe were selected by the acquisition of tetracycline resistance on 707-FLPe at 30°C. The expression of the FLPe recombination protein was induced by a temperature shift to 37°C. Through the induction, the FRT sites, which had disrupted the target gene, were recombined and the kanamycin selection marker cassette was lost. The clones of interest grew on LB agar plates but not on LB agar plates containing tetracycline or kanamycin. The Km^s and Tet^s clones with gene disruption were verified by colony PCR followed by DNA sequencing.

Competition experiments. *E. coli* W3110 and the three ghost-derived mutator strains (g62, gw25, gb37) were grown overnight in LB broth and the resulting cultures spread onto LB agar containing 100 µg mL^{-1} rifampicin or 10 µg mL^{-1} nalidixic acid. Thirty rifampicin-resistant mutants and nalidixic acid mutants derived from ghost-derived mutator mutant were then randomly chosen per

isolate. In order to identify the mutation in *rpoB* responsible for rifampicin resistance and *gyrA* responsible for nalidixic acid in individual mutants, each antibiotic resistance target gene was amplified and then sequenced. The primers used were as follows: to amplify part of the *rpoB* gene, 5’-CGTCGTATCCGTTCCGTTGG-3’ and 5’-TTCACCCGGATAACATCTCGTC-3’ ; and to amplify the *gyrA* gene, 5’-TACACCGGTCCACATTGAGG-3’ and 5’-TTAATGATTGCCGCCGTCGG-3’. Each resistant clone was grown from a single colony in LB broth supplemented with the respective antibiotic and stored in 15% glycerol at −80°C.

The pairs of W3110 and W3110 strain, W3110 and g62 strain, W3110 and gw25, W3110 and gb37 that carried identical gyrA or rpoB mutation, were subjected to competition experiments because different nalidixic acid/rifampicin resistance mutations have specific fitness benefits (Katz and Hershberg, 2013.). Each resistant clone contained the following amino acid substitution; H526Y on rpoB and D87G on gyrA for W3110 and g62 pair; H526Y on rpoB and S83L on gyrA for W3110 and gw25 pair; H526Y on rpoB and A119E on gyrA for W3110 and gb37 pair; H526Y on rpoB and D87G or S83L or A119E on gyrA for W3110 and W3110 pair.

About 30,000–60,000 unmarked W3110 cells were spotted on a sterile polycarbonate filter membrane. Since mutant frequencies of nalidixic acid and rifampicin resistance in W3110 were $<10^{-8}$, no Rif^R or Nal^R mutants were present in the initial inoculum. Then a mixture of 100–200 marked W3110 cells (marked with either Nal^R or Rif^R) and 100–200 marked ghost-derived mutator cells (marked with either Rif^R or Nal^R) were spotted on the same filter.

The exact ratio of unmarked, nalidixic acid- or rifampicin- resistant cells inoculated onto the filter was estimated through live counts (day 0). Then the ratio of marked ghost-derived mutator to marked W3110 was measured after 2 days. The measurement of these ratios was done by scraping the mixture spots, placing them in PBS. Then 50 μl from the suspension were plated on LB agar containing 100 μg mL^{-1} rifampicin or 10 μg mL^{-1} nalidixic acid, and viable cells were counted the following day.

In order to control for possible effects of the antibiotic marker themselves on fitness, we conducted same competition experiments between Rif^R- and Nal^R- marked W3110 cells. The results with the marked W3110 cells were used to normalize the measured competitive index (CI) for Rif^R-marked mutator versus Nal^R-marked W3110 strains. Three replicates of each competition experiment were performed. The CI was calculated for each Rif^R or Nal^R -marked ghost-derived mutator from the change in the Nal^R or Rif^R /W3110 ratio from Day 0 to Day 2. During 2 days, the inoculated cells increased 10^4 to 10^5-fold.

References

Katz S, Hershberg R. 2013. Elevated mutagenesis does not explain the increased frequency of antibiotic resistant mutants in starved aging colonies. *PLoS Genet* **9**:e1003968.

2 Supplementary Figures and Tables

2.1 Supplementary Figures

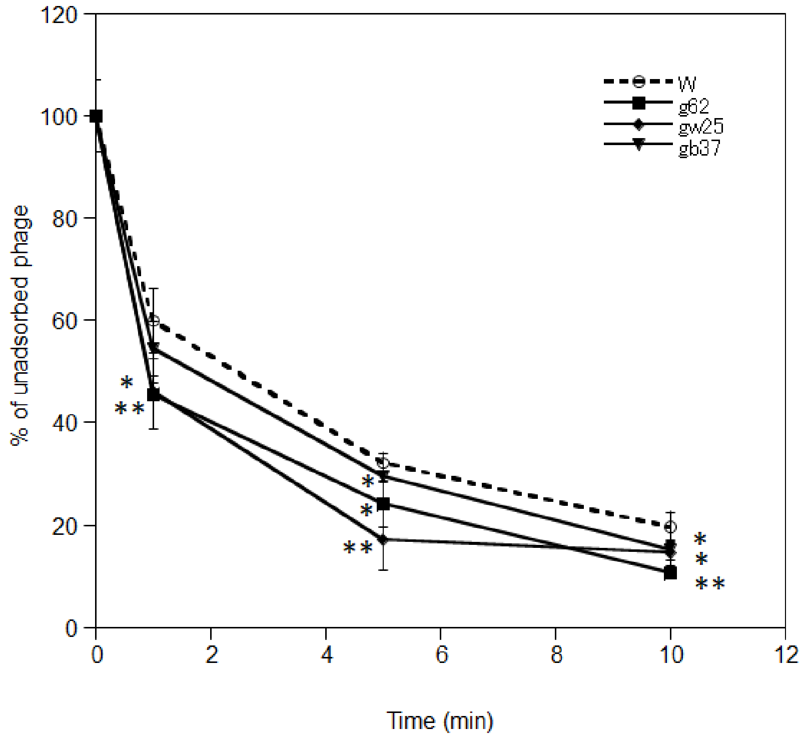

Supplementary Figure 1. Phage adsorption assay with mutator strains derived from ghost infection. E. coli was grown to the mid-log phase in LB broth (pH 7.0). Phage T4 was adsorbed to the cells in LB broth and incubated at 37°C. Data are the means from three independent experiments. Error bars represent standard deviations of the mean. The asterisk indicates statistical significance (*$P < 0.05$, **$P < 0.01$) compared with the W3110 parental strain (W) treated under same conditions.

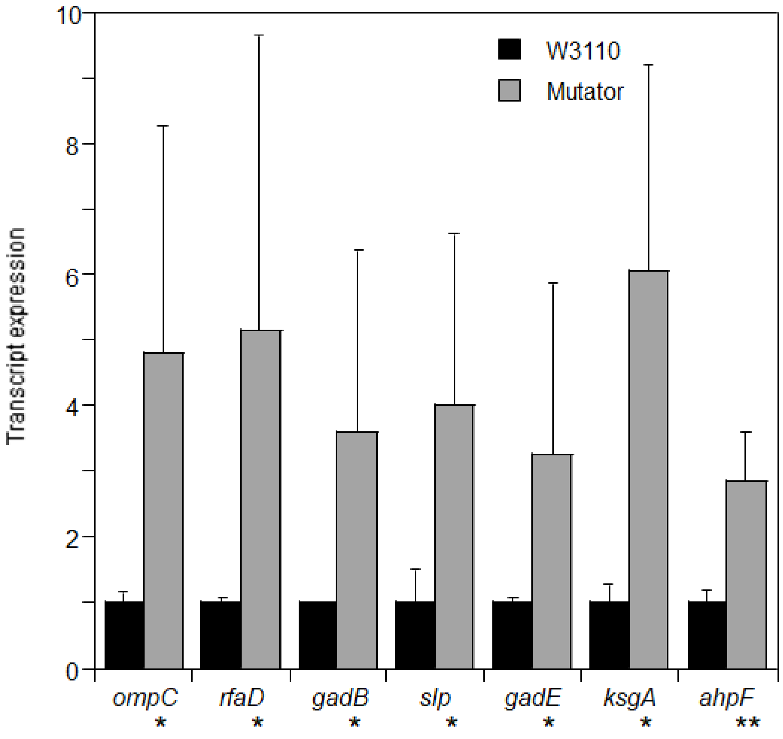

Supplementary Figure 2. Relative mRNA levels for selected upregulated genes involved in stress resistance in three ghost-derived mutator strains. qRT-PCR was performed with mutator strains and the W3110 RNA as a template. Average mRNA level ratios in three ghost-derived mutator strains are given under each gene. The asterisk indicates statistically significant (*P<0.05, **P<0.01) compared with the W3110 parental strain treated with the same condition.

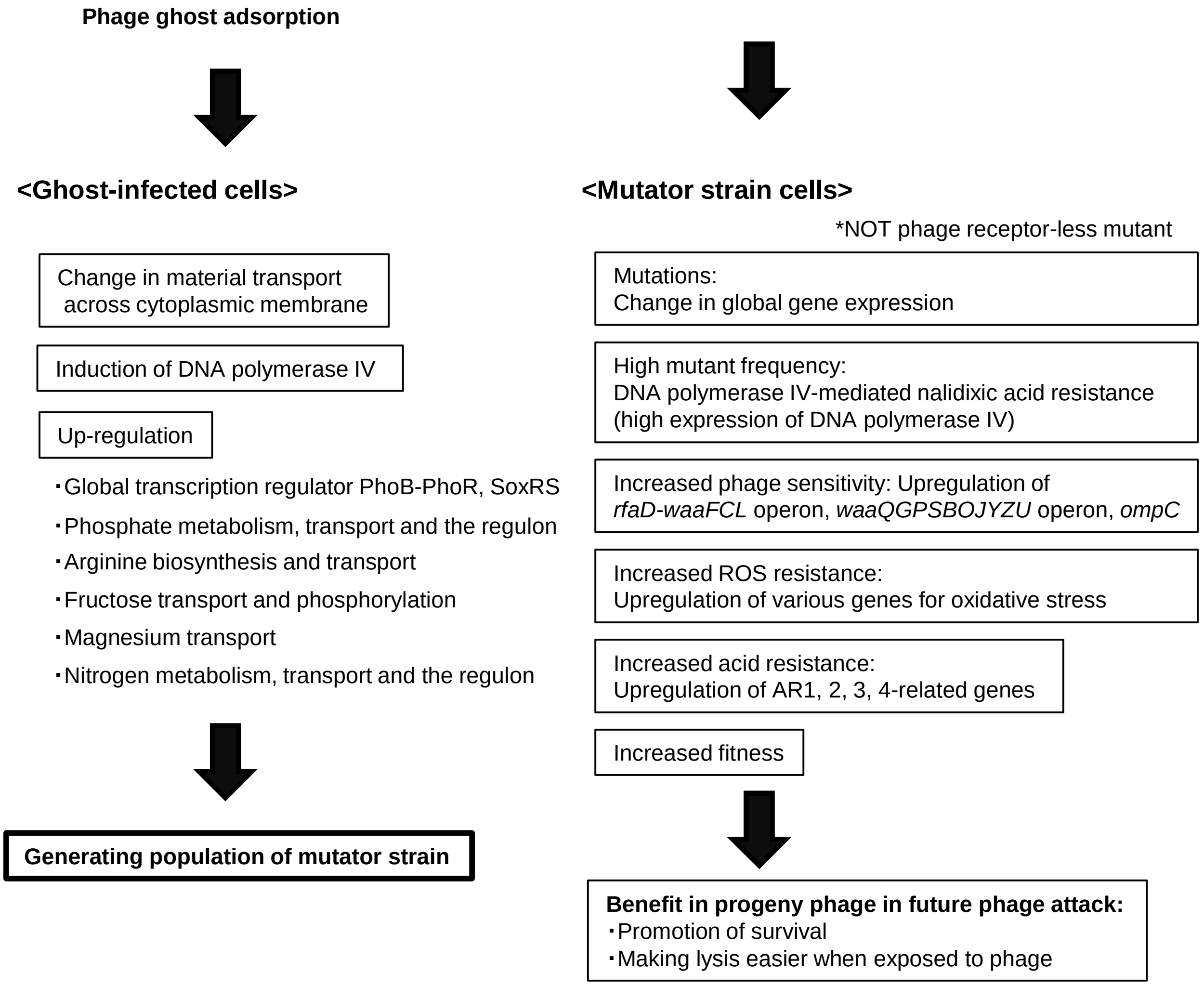

Supplementary Figure 3. Summary of the response of host cells after ghost adsorption.

2.2 Supplementary Tables

Supplementary Table 1. Primers used for quantitative reverse transcription PCR analysis.

Gene	Primer	Sequence (5’-3’)
dinB	dinB659f	gcattttgtgggagcgtagt
	dinB897r	ggtggtttgctgaaaatcgt
ompC	ompC165f	ctacatgcgtcttggcttca
	ompC308r	aatttcagacctgcgaatgc
rfaD	rfaD470f	aagcgaactcgcagattgtt
	rfaD635r	acgaagtcgcgtttgaagtt
gadB	gadB1038f	ttaccaggttgccgcttatc
	gadB1200r	acgcagacgttcagagaggt
slp	slp357f	gggtgaacaacctggcttta
	slp490r	atgcaccatagccgtaatcc
gadE	gadE34f	ttacagggcttttggcagtt
	gadE202r	tctcggcatctaatttctcca
ksgA	ksgA648f	agcctttaaccagcgtcgta
	ksgA802r	cgttctccgccagatagttc
ahpF	ahpF730f	cgttttggtggtcagatcct
	ahpF893r	actgctgctgggatcagttt
16S rRNA	16S-rtF	gtgcaatattccccactgct
	16S-rtR	cgatccctagctggtctgag

Supplementary Table 2. Summary of base substitutions on *gyrA* in mutator strains.

	Mutant frequency (x 10^{-9})		
Base substitutions	Wild[a]	Mutator[a]	Ratio[b]
G:C to A:T	0.30	23	77
A:T to G:C	0.27	ND[c]	<4.1
A:T to T:A	ND	ND	
G:C to T:A	0.33	34	103
G:C to C:G	0.30	ND	<3.7
A:T to C:G	ND	96	>640

[a] The numbers indicated in columns are the means from three independent experiments.

[b] Ratio of mutant frequency in the mutator strains to that in the parent strainW3110.

[c] ND, Not detected.

Supplementary Table 3. Relative mRNA levels for upregulated operons in ghost infected *E. coli* W3110

Gene Designation	Gene Name	Protein function	mRNA*	Z score	p-value	Ratio to phage
b0010	*yaaH*	hypothetical protein	3.34	2.10	0.035	5.01
b0032	*carA*	carbamoyl-phosphate synthetase, glutamine	3.20	2.03	0.043	4.03
b0033	*carB*	carbamoyl-phosphate synthase large subunit	3.29	2.08	0.038	4.70
b0161	*degP*	periplasmic serine protease Do; heat shock protein HtrA	5.80	3.09	0.002	6.61
b0241	*phoE*	outer membrane pore protein E	6.53	3.30	0.001	1.27
b0273	*argF*	ornithine carbamoyltransferase 2, chain F	7.68	3.59	0.000	4.82
b0288	*ykgJ*	putative ferredoxin	3.78	2.32	0.020	0.73
b0383	*phoA*	alkaline phosphatase	6.08	3.17	0.002	1.21
b0384	*psiF*	induced by phosphate starvation	9.30	3.94	0.000	1.28
b0399	*phoB*	positive response regulator for pho regulon, sensor is PhoR	21.69	5.45	0.000	5.23
b0400	*phoR*	positive and negative sensor protein for pho regulon	18.37	5.16	0.000	5.15
b0593	*entC*	isochorismate hydroxymutase 2, enterochelin biosynthesis	2.45	1.55	0.121	2.31
b0594	*entE*	2,3-dihydroxybenzoate-AMP ligase	4.77	2.74	0.006	4.29
b0595	*entB*	2,3-dihydro-2,3-dihydroxybenzoate synthetase, isochroismatase	6.94	3.41	0.001	4.95
b0596	*entA*	2,3-dihydro-2,3-dihydroxybenzoate dehydrogenase, enterochelin biosynthesis	7.50	3.55	0.000	5.00
b0597	*ybdB*	hypothetical protein	5.38	2.96	0.003	3.80
b0699	*ybfA*	hypothetical protein	4.50	2.64	0.008	1.23
b0781	*moaA*	molybdopterin biosynthesis, protein A	3.92	2.39	0.017	1.96
b0782	*moaB*	molybdopterin biosynthesis, protein B	5.56	3.02	0.003	2.39
b0783	*moaC*	molybdopterin biosynthesis, protein C	4.49	2.63	0.008	2.13
b0784	*moaD*	molybdopterin biosynthesis	4.02	2.43	0.015	2.17
b0785	*moaE*	molybdopterin converting factor, subunit 2	4.15	2.49	0.013	2.21
b0802	*ybiJ*	hypothetical protein	3.58	2.23	0.026	0.86
b0836	*bssR*	putative receptor	10.73	4.19	0.000	2.16
b0860	*artJ*	arginine 3rd transport system periplasmic binding protein	9.31	3.94	0.000	7.00
b0861	*artM*	arginine 3rd transport system permease protein	4.16	2.49	0.013	9.98
b0862	*artQ*	arginine 3rd transport system permease protein	4.07	2.46	0.014	9.33
b0863	*artI*	arginine 3rd transport system periplasmic binding protein	4.96	2.81	0.005	8.70
b0864	*artP*	ATP-binding component of 3rd arginine transport system	4.45	2.62	0.009	9.92
b0953	*rmf*	ribosome modulation factor	7.44	3.54	0.000	2.08
b1020	*phoH*	PhoB-dependent, ATP-binding pho regulon component; may be helicase; induced by P starvation	3.45	2.16	0.031	1.17
b1112	*bhsA*	hypothetical protein (biofilm)	8.21	3.71	0.000	2.11
b1221	*narL*	pleiotrophic regulation of anaerobic respiration: response regulator for nar, frd, dms and tor genes	4.55	2.66	0.008	2.99
b1222	*narX*	nitrate/nitrate sensor, histidine protein kinase acts on NarL regulator	3.90	2.38	0.017	2.66
b1223	*narK*	nitrite extrusion protein	14.46	4.73	0.000	7.52
b1224	*narG*	nitrate reductase 1, alpha subunit	12.04	4.40	0.000	3.91
b1225	*narH*	nitrate reductase 1, beta subunit	12.72	4.50	0.000	3.40
b1226	*narJ*	nitrate reductase 1, delta subunit, assembly function	9.05	3.89	0.000	2.36
b1227	*narI*	nitrate reductase 1, cytochrome b	1.79	0.99	0.324	1.01
b1380	*ldhA*	fermentative D-lactate dehydrogenase, NAD-dependent	3.96	2.41	0.016	2.36
b1452	*yncE*	putative receptor	3.90	2.38	0.017	1.56
b1528	*ydeA*	putative resistance / regulatory protein	4.48	2.63	0.009	3.06
b1541	*ydfZ*	hypothetical protein	4.10	2.47	0.014	1.74
b1684	*sufA*	hypothetical protein	3.16	2.00	0.045	0.80
b1796	*yoaG*	hypothetical protein	3.01	1.92	0.055	0.93
b1797	*yeaR*	hypothetical protein	4.27	2.54	0.011	1.34
b1967	*hchA*	hypothetical protein	3.43	2.15	0.031	1.43
b2069	*yegD*	putative heat shock protein	3.22	2.04	0.042	3.20
b2141	*yohJ*	hypothetical protein	8.64	3.80	0.000	2.07
b2142	*yohK*	putative seritonin transporter	8.94	3.87	0.000	2.67
b2167	*fruA*	PTS system, fructose-specific transport protein	2.13	1.30	0.194	0.38
b2168	*fruK*	fructose-1-phosphate kinase	3.27	2.07	0.039	0.33
b2169	*fruB*	PTS system, fructose-specific IIA/fpr component	4.25	2.53	0.011	0.39
b2419	*yfeK*	hypothetical protein	4.76	2.74	0.006	3.27
b2420	*yfeS*	hypothetical protein	2.68	1.71	0.088	2.31
b2592	*clpB*	heat shock protein	5.04	2.84	0.005	3.45
b2670	*ygaW*	hypothetical protein	3.32	2.09	0.036	2.55
b2818	*argA*	N-acetylglutamate synthase; amino acid acetyltransferase	4.80	2.75	0.006	3.19
b2924	*mscS*	putative transport protein	3.95	2.40	0.016	3.00
b3172	*argG*	argininosuccinate synthetase	4.37	2.58	0.010	5.13

b3364	*tsgA*	putative transport	6.16	3.20	0.001	3.94
b3365	*nirB*	nitrite reductase	11.05	4.25	0.000	2.90
b3366	*nirD*	nitrite reductase	12.10	4.41	0.000	2.63
b3367	*nirC*	nitrite reductase activity	6.73	3.36	0.001	3.00
b3368	*cysG*	uroporphyrinogen III methylase; sirohaeme biosynthesis	2.96	1.89	0.059	2.88
b3686	*ibpB*	heat shock protein	8.83	3.84	0.000	1.01
b3687	*ibpA*	heat shock protein	6.05	3.17	0.002	1.04
b3724	*phoU*	negative regulator for pho regulon and putative enzyme in phosphate metabolism	11.72	4.35	0.000	5.13
b3725	*pstB*	ATP-binding component of high-affinity phosphate-specific transport system	14.51	4.73	0.000	6.29
b3726	*pstA*	high-affinity phosphate-specific transport system	16.24	4.94	0.000	6.61
b3727	*pstC*	high-affinity phosphate-specific transport system, cytoplasmic membrane component	19.17	5.23	0.000	7.89
b3728	*pstS*	high-affinity phosphate-specific transport system; periplasmic phosphate-binding protein	32.68	6.19	0.000	7.58
b3908	*sodA*	superoxide dismutase, manganese	3.11	1.98	0.048	4.41
b3958	*argC*	N-acetyl-gamma-glutamylphosphate reductase	12.79	4.51	0.000	7.14
b3959	*argB*	acetylglutamate kinase	5.88	3.12	0.002	6.77
b3960	*argH*	argininosuccinate lyase	6.67	3.34	0.001	7.06
b4030	*psiE*	phosphate starvation-inducible protein	3.53	2.20	0.028	0.64
b4060	*yjcB*	hypothetical protein	7.21	3.48	0.001	2.29
b4242	*mgtA*	Mg2+ transport ATPase, P-type 1	4.51	2.64	0.008	0.44
b4254	*argI*	ornithine carbamoyltransferase 1	7.92	3.65	0.000	4.92

* Relative mRNA level was shown as average of those in three independent experiments.

Supplementary Table 4. Relative mRNA levels for downregulated operons in ghost infected *E. coli* W3110

Gene						
Designation	Name	Protein function	mRNA*	Z score	p-value	Ratio to phage
b0080	*fruR(Cra)*	transcriptional repressor of fru operon and others	0.28	-2.36	0.018	0.83
b0268	*yagE*	putative lyase/synthase	0.24	-2.58	0.010	0.65
b0269	*yagF*	putative dehydratase	0.39	-1.74	0.082	0.71
b0316	*yahB*	putative transcriptional regulator LYSR-type	0.34	-2.00	0.046	0.68
b0411	*tsx*	nucleoside channel; receptor of phage T6 and colicin K	0.19	-3.04	0.002	0.49
b0553	*nmpC*	outer membrane porin protein; locus of qsr prophage	0.21	-2.82	0.005	0.41
b0612	*citT*	putative a membrane protein	0.23	-2.71	0.007	1.33
b0613	*citG*	hypothetical protein	0.20	-2.90	0.004	1.62
b0614	*citX*	hypothetical protein	0.26	-2.45	0.014	1.20
b0615	*citF*	citrate lyase alpha chain	0.22	-2.79	0.005	1.66
b0616	*citE*	citrate lyase beta chain	0.20	-2.91	0.004	1.18
b0617	*citD*	citrate lyase acyl carrier protein	0.22	-2.73	0.006	1.31
b0618	*citC*	citrate lyase synthetase	0.17	-3.26	0.001	0.59
b0651	*rihA*	putative tRNA synthetase	0.15	-3.43	0.001	0.38
b0899	*ycaM*	putative transport	0.15	-3.44	0.001	0.41
b0929	*ompF*	outer membrane protein 1a	0.31	-2.17	0.030	0.62
b0963	*mgsA*	methylglyoxal synthase	0.30	-2.21	0.027	0.62
b0972	*hyaA*	hydrogenase-1 small subunit	0.27	-2.43	0.015	1.30
b0973	*hyaB*	hydrogenase-1 large subunit	0.34	-1.99	0.047	1.67
b0974	*hyaC*	probable Ni/Fe-hydrogenase 1 b-type cytochrome subunit	0.37	-1.83	0.068	1.75
b0975	*hyaD*	processing of HyaA and HyaB proteins	0.38	-1.77	0.077	1.33
b0976	*hyaE*	processing of HyaA and HyaB proteins	0.36	-1.90	0.058	1.36
b0977	*hyaF*	nickel incorporation into hydrogenase-1 proteins	0.32	-2.12	0.034	1.25
b1329	*mppA*	putative transport periplasmic protein	0.33	-2.04	0.041	0.73
b1421	*trg*	methyl-accepting chemotaxis protein III, ribose sensor receptor	0.30	-2.22	0.026	0.66
b1587	*ynfE*	putative oxidoreductase, major subunit	0.44	-1.53	0.126	0.48
b1588	*ynfF*	putative oxidoreductase, major subunit	0.24	-2.63	0.008	0.78
b1589	*ynfG*	putative oxidoreductase, Fe-S subunit	0.26	-2.50	0.013	0.79
b1590	*ynfH*	putative DMSO reductase anchor subunit	0.23	-2.73	0.006	0.86
b1591	*dmsD*	putative oxidoreductase component	0.39	-1.75	0.080	1.16
b1594	*dgsA*	putative NAGC-like transcriptional regulator	0.30	-2.21	0.027	0.66
b1799	*yeaT*	putative transcriptional regulator LYSR-type	0.16	-3.31	0.001	0.53
b1823	*cspC*	cold shock protein	0.33	-2.05	0.041	0.77
b1853	*yebK*	hypothetical protein	0.34	-1.98	0.048	0.71
b1904	*yecR*	hypothetical protein	0.30	-2.19	0.028	1.26
b1976	*mtfA*	hypothetical protein	0.25	-2.54	0.011	0.50
b2091	*gatD*	galactitol-1-phosphate dehydrogenase	0.30	-2.20	0.028	0.78
b2095	*gatZ*	putative tagatose 6-phosphate kinase 1	0.35	-1.92	0.055	0.93
b2096	*gatY*	tagatose-bisphosphate aldolase 1	0.32	-2.09	0.036	0.76
b2238	*yfaH*	hypothetical protein	0.28	-2.34	0.019	0.31
b2239	*glpQ*	glycerophosphodiester phosphodiesterase, periplasmic	0.24	-2.60	0.009	0.38
b2240	*glpT*	sn-glycerol-3-phosphate permease	0.22	-2.75	0.006	0.37
b2244	*yfaD*	hypothetical protein	0.29	-2.27	0.023	0.43
b2272	*yfbM*	hypothetical protein	0.26	-2.46	0.014	0.48
b2398	*yfeC*	hypothetical protein	0.28	-2.37	0.018	0.80
b2399	*yfeD*	hypothetical protein	0.34	-1.97	0.049	0.81
b2426	*ucpA*	putative oxidoreductase	0.25	-2.54	0.011	0.84
b2702	*srlA*	PTS system, glucitol/sorbitol-specific IIC component, one of two	0.15	-3.42	0.001	1.11
b2703	*srlE*	PTS system, glucitol/sorbitol-specific IIB component and second of two IIC components	0.19	-3.04	0.002	0.88
b2704	*srlB*	PTS system, glucitol/sorbitol-specific enzyme IIA component	0.28	-2.31	0.021	2.32
b2705	*srlD*	glucitol	0.30	-2.19	0.029	1.31
b2725	*hycA*	transcriptional repression of hyc and hyp operons	0.33	-2.03	0.042	1.43
b2795	*ygdH*	hypothetical protein	0.31	-2.17	0.030	0.95
b2796	*sdaC*	probable serine transporter	0.22	-2.75	0.006	1.05
b2797	*sdaB*	L-serine dehydratase	0.20	-2.98	0.003	1.14
b2798	*ygdG*	5-3 exonuclease	0.42	-1.60	0.109	1.42
b2869	*ygeV*	putative transcriptional regulator	0.30	-2.21	0.027	0.62
b2956	*yggM*	putative alpha helix chain	0.34	-1.97	0.049	0.52
b2957	*ansB*	periplasmic L-asparaginase II	0.34	-2.01	0.044	0.73
b2964	*nupG*	transport of nucleosides, permease protein	0.17	-3.25	0.001	0.47

b2990	*hybG*	hydrogenase-2 operon protein: may effect maturation of large subunit of hydrogenase-2	0.29	-2.30	0.021	1.28
b2991	*hybF*	may modulate levels of hydrogenease-2	0.28	-2.35	0.019	1.40
b2992	*hybE*	member of hyb operon	0.31	-2.14	0.032	1.44
b2993	*hybD*	probable processing element for hydrogenase-2	0.28	-2.31	0.021	1.36
b2994	*hybC*	probable large subunit, hydrogenase-2	0.28	-2.36	0.018	1.58
b2995	*hybB*	probable cytochrome Ni/Fe component of hydrogenase-2	0.32	-2.09	0.037	1.40
b2996	*hybA*	hydrogenase-2 small subunit	0.33	-2.05	0.041	1.03
b2997	*hybO*	putative hydrogenase subunit	0.29	-2.27	0.023	1.16
b3020	*ygiS*	putative transport periplasmic protein	0.23	-2.66	0.008	0.56
b3072	*aer*	aerotaxis sensor receptor, flavoprotein	0.33	-2.04	0.042	0.82
b3074	*ygjH*	putative tRNA synthetase	0.25	-2.55	0.011	0.63
b3076	*ebgA*	evolved beta-D-galactosidase, alpha subunit; cryptic gene	0.22	-2.75	0.006	0.58
b3077	*ebgC*	evolved beta-D-galactosidase, beta subunit; cryptic gene	0.16	-3.34	0.001	0.57
b3078	*ygjI*	putative oxidoreductase	0.15	-3.44	0.001	0.75
b3112	*tdcG*	putative L-serine dehydratase	0.14	-3.57	0.000	2.06
b3113	*tdcF*	hypothetical protein	0.17	-3.28	0.001	2.56
b3114	*tdcE*	probable formate acetyltransferase 3	0.15	-3.45	0.001	1.70
b3115	*tdcD*	putative kinase	0.17	-3.19	0.001	1.19
b3116	*tdcC*	anaerobically inducible L-threonine, L-serine permease	0.20	-2.93	0.003	0.91
b3117	*tdcB*	threonine dehydratase, catabolic	0.09	-4.45	0.000	0.52
b3118	*tdcA*	transcriptional activator of tdc operon	0.07	-4.92	0.000	0.44
b3263	*yhdU*	hypothetical protein	0.33	-2.02	0.043	0.77
b3415	*gntT*	high-affinity transport of gluconate / gluconate permease	0.25	-2.52	0.012	0.60
b3416	*malQ*	4-alpha-glucanotransferase	0.34	-2.00	0.045	1.33
b3417	*malP*	maltodextrin phosphorylase	0.17	-3.24	0.001	0.73
b3418	*malT*	positive regulator of mal regulon	0.17	-3.23	0.001	0.45
b3426	*glpD*	sn-glycerol-3-phosphate dehydrogenase	0.30	-2.19	0.029	0.64
b3436	*gntU*	low-affinity gluconate transport permease protein, interrupted	0.30	-2.19	0.028	0.73
b3528	*dctA*	uptake of C4-dicarboxylic acids	0.26	-2.47	0.014	0.98
b3547	*yhjX*	putative resistance protein	0.10	-4.22	0.000	0.17
b3748	*rbsD*	D-ribose high-affinity transport system; membrane-associated protein	0.30	-2.23	0.026	0.75
b3749	*rbsA*	ATP binding component of D-ribose high-affinity transport system	0.19	-3.07	0.002	0.73
b3750	*rbsC*	D-ribose high-affinity transport system	0.21	-2.82	0.005	0.92
b3751	*rbsB*	D-ribose periplasmic binding protein	0.38	-1.81	0.071	1.04
b3752	*rbsK*	ribokinase	0.28	-2.35	0.019	0.90
b3753	*rbsR*	regulator for rbs operon	0.26	-2.47	0.014	0.75
b3832	*rmuC*	putative alpha helix chain	0.20	-2.95	0.003	0.63
b3872	*yihL*	putative transcriptional regulator	0.18	-3.09	0.002	0.67
b3873	*yihM*	hypothetical protein	0.17	-3.20	0.001	0.40
b3926	*glpK*	glycerol kinase	0.19	-3.00	0.003	0.61
b3927	*glpF*	facilitated diffusion of glycerol	0.16	-3.35	0.001	0.65
b3934	*cytR*	regulator for deo operon, udp, cdd, tsx, nupC, and nupG	0.22	-2.79	0.005	0.59
b4032	*malG*	part of maltose permease, inner membrane	0.25	-2.56	0.010	0.78
b4033	*malF*	part of maltose permease, periplasmic	0.08	-4.54	0.000	0.71
b4034	*malE*	periplasmic maltose-binding protein; substrate recognition for transport and chemotaxis	0.05	-5.47	0.000	1.10
b4035	*malK*	ATP binding component of transport system for maltose	0.01	-7.60	0.000	1.01
b4036	*lamB*	phage lambda receptor protein; maltose high-affinity receptor	0.05	-5.37	0.000	1.33
b4037	*malM*	periplasmic protein of mal regulon	0.06	-5.05	0.000	0.90
b4055	*aphA*	diadenosine tetraphosphatase	0.14	-3.54	0.000	0.34
b4118	*melR*	regulator of melibiose operon	0.18	-3.13	0.002	0.77
b4122	*fumB*	fumarate hydratase Class I; anaerobic isozyme	0.19	-3.01	0.003	0.67
b4123	*dcuB*	anaerobic dicarboxylate transport	0.15	-3.41	0.001	0.48
b4124	*dcuR*	putative 2-component transcriptional regulator	0.34	-1.98	0.048	0.88
b4125	*dcuS*	putative 2-component sensor protein	0.28	-2.31	0.021	0.80
b4139	*aspA*	aspartate ammonia-lyase	0.31	-2.13	0.033	0.87
b4188	*yjfN*	hypothetical protein	0.20	-2.95	0.003	0.75
b4189	*yjfO*	hypothetical protein	0.15	-3.40	0.001	0.53
b4193	*ulaA*	hypothetical protein	0.16	-3.34	0.001	0.72
b4194	*ulaB*	hypothetical protein	0.20	-2.93	0.003	0.62
b4195	*ulaC*	putative PTS system enzyme II A component	0.21	-2.85	0.004	0.94
b4196	*ulaD*	probable hexulose-6-phosphate synthase	0.22	-2.78	0.005	0.82
b4197	*ulaE*	putative hexulose-6-phosphate isomerase	0.27	-2.38	0.017	0.92
b4198	*ulaF*	putative epimerase/aldolase	0.20	-2.91	0.004	0.88

b4239	*treC*	trehalase 6-P hydrolase	0.17	-3.21	0.001	0.58
b4240	*treB*	PTS system enzyme II, trehalose specific	0.19	-3.01	0.003	0.64
b4307	*yjhQ*	hypothetical protein	0.33	-2.07	0.039	0.69
b4322	*uxuA*	mannonate hydrolase	0.20	-2.96	0.003	0.65
b4323	*uxuB*	D-mannonate oxidoreductase	0.26	-2.45	0.014	0.83
b4342	*yjiT*	hypothetical protein	0.31	-2.15	0.032	0.55
b4352	*yjiA*	hypothetical protein	0.22	-2.76	0.006	0.53
b4353	*yjiX*	hypothetical protein	0.18	-3.09	0.002	0.44
b4354	*yjiY*	putative carbon starvation protein	0.18	-3.10	0.002	0.41
b4357	*yjjM*	hypothetical protein	0.34	-2.00	0.045	0.58

* Relative mRNA level was shown as average of those in three independent experiments.
